# Type IV Collagen Controls the Axogenesis of Cerebellar Granule Cells by Regulating Basement Membrane Integrity in Zebrafish

**DOI:** 10.1371/journal.pgen.1005587

**Published:** 2015-10-09

**Authors:** Miki Takeuchi, Shingo Yamaguchi, Shigenobu Yonemura, Kisa Kakiguchi, Yoshikatsu Sato, Tetsuya Higashiyama, Takashi Shimizu, Masahiko Hibi

**Affiliations:** 1 Laboratory of Organogenesis and Organ Function, Bioscience and Biotechnology Center, Nagoya University, Nagoya, Aichi, Japan; 2 Devision of Biological Science, Graduate School of Science, Nagoya University, Nagoya, Aichi, Japan; 3 Ultrastructural Research Team, RIKEN Center for Life Science Technologies, Kobe, Hyogo, Japan; 4 Institute of Transformative Bio-Molecules, Nagoya University, Nagoya, Aichi, Japan; Fred Hutchinson Cancer Research Center, UNITED STATES

## Abstract

Granule cells (GCs) are the major glutamatergic neurons in the cerebellum, and GC axon formation is an initial step in establishing functional cerebellar circuits. In the zebrafish cerebellum, GCs can be classified into rostromedial and caudolateral groups, according to the locations of their somata in the corresponding cerebellar lobes. The axons of the GCs in the caudolateral lobes terminate on crest cells in the dorsal hindbrain, as well as forming en passant synapses with Purkinje cells in the cerebellum. In the zebrafish mutant *shiomaneki*, the caudolateral GCs extend aberrant axons. Positional cloning revealed that the *shiomaneki* (*sio*) gene locus encodes Col4a6, a subunit of type IV collagen, which, in a complex with Col4a5, is a basement membrane (BM) component. Both *col4a5* and *col4a6* mutants displayed similar abnormalities in the axogenesis of GCs and retinal ganglion cells (RGCs). Although type IV collagen is reported to control axon targeting by regulating the concentration gradient of an axonal guidance molecule Slit, Slit overexpression did not affect the GC axons. The structure of the BM surrounding the tectum and dorsal hindbrain was disorganized in the *col4a5* and *col4a6* mutants. Moreover, the abnormal axogenesis of the caudolateral GCs and the RGCs was coupled with aberrant BM structures in the type IV collagen mutants. The regrowth of GC axons after experimental ablation revealed that the original and newly formed axons displayed similar branching and extension abnormalities in the *col4a6* mutants. These results collectively suggest that type IV collagen controls GC axon formation by regulating the integrity of the BM, which provides axons with the correct path to their targets.

## Author Summary

The cerebellum is involved in motor coordination and motor learning. Granule cells are the major excitatory neurons in the cerebellum. It is largely unknown how the formation of cerebellar neural circuits, including the elaboration of granule cell axons, is controlled. We investigated a zebrafish mutant *shiomaneki*, in which some of the granule cells have abnormal axons. We identified *collagen* (*col*) *4a6* as the gene responsible for the mutant phenotype. Col4a6 forms a complex with Col4a5, which is a component of the basement membrane. We found that mutants of both *col4a5* and *col4a6* showed similar axonal abnormalities in both the granule cells and the retinal ganglion cells, and that the basement membrane structure surrounding the central nervous system was disrupted in these mutants. Furthermore, the abnormalities in granule cell axon formation were coupled with aberrant basement membrane structures in the *col4a6* mutants. These data suggest that type IV collagen controls the axon formation of some types of neurons by establishing and/or maintaining the integrity of the basement membrane, which provides axons with the correct path to their targets. These findings may explain some aspects of a human disorder, Alport syndrome, which is caused by mutations in type IV collagen genes.

## Introduction

**Fig 1:**
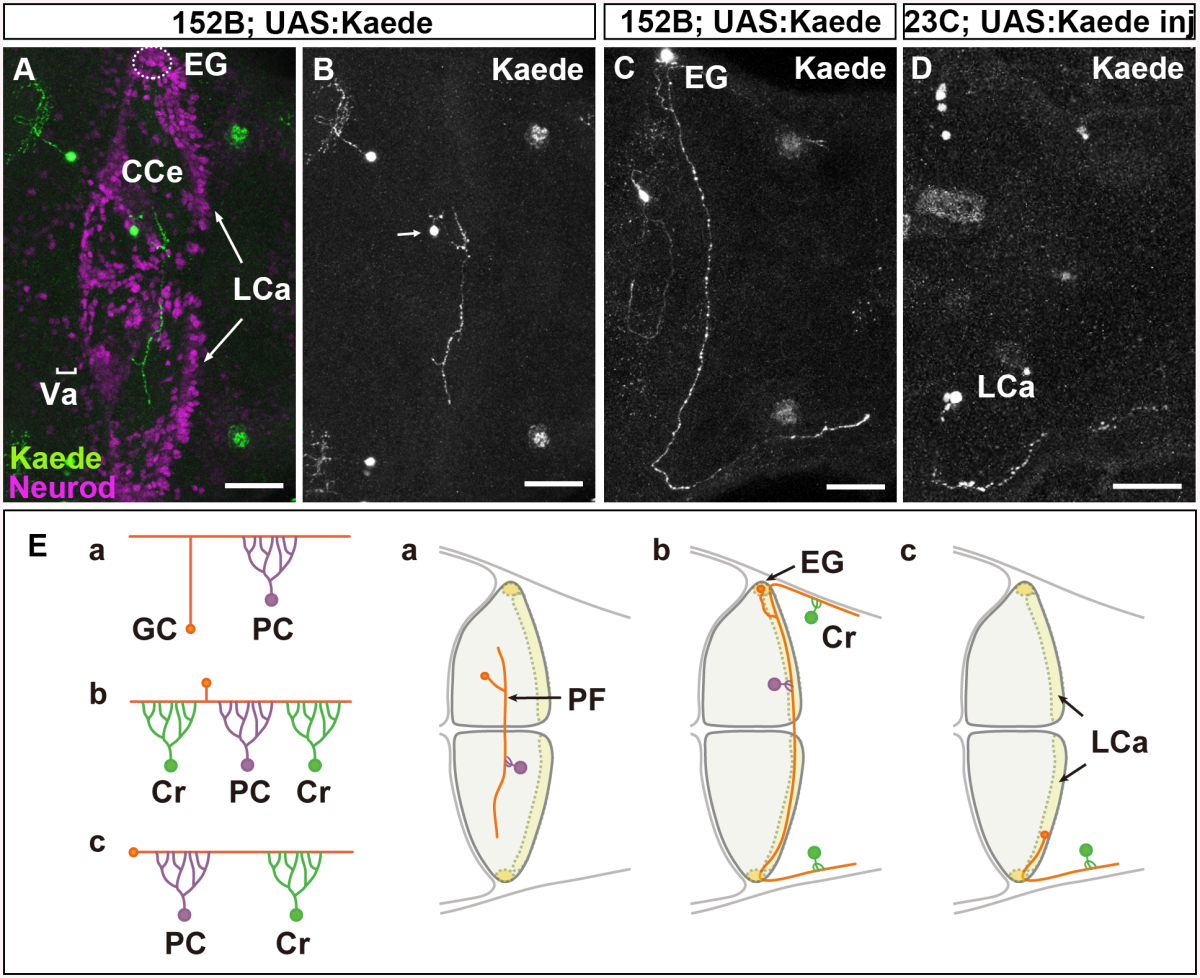
There are three types of GCs in zebrafish cerebellum. (A-D) Single-cell labeling was performed by crossing the GC-specific Gal4 line gSA2AzGFF152B and partially silenced UAS:Kaede reporter line (A, B, C), or by injecting UAS:Kaede reporter DNA in a Tol1 vector and Tol1 transposase RNA into the GC (in the LCa)-specific Gal4 line gSAIGFF23C (Fig 1D). Larvae at 5 dpf that expressed Kaede in one or a few GCs were selected, and Kaede was visualized by immunostaining with an anti-Kaede antibody. Dorsal projection views in the cerebellum. Co-staining with an antibody against Neurod, a GC marker, indicated that the Kaede^+^ cells in the cerebellum were GCs (A). (E) Schematic representation of the GC types. The GCs in the Va and CCe (rostromedial lobes) had a T-shaped axon, which formed parallel fibers with other GC axons, and targeted the dendrites of PCs (Ea). The GCs in the EG also had a T-shaped axon, which extended bilaterally and turned caudally to the dorsal hindbrain (Eb). The GCs in the LCa extended their axon only ipsilaterally, and it turned caudally. The GCs in the EG and LCa (caudolateral lobes) made synapses on the dendrites of PCs in the cerebellum and on those of crest cells (Cr) in the dorsal hindbrain (crista cerebellaris). Scale bars: 40 μm in A-D.

The cerebellum is involved in various brain functions, including motor coordination and motor learning [1–3]. Since the structure of the cerebellum is basically conserved among vertebrates [4,5], the zebrafish cerebellum provides a good model for understanding cerebellar development and functioning [6–8]. In both the mammalian and zebrafish cerebellum, granule cells (GCs) are the major glutamatergic neurons. The teleost cerebellum contains at least two different types of GCs (Fig 1) that have different locations and developmental processes and contribute to distinct neural circuits [9–11].

The GCs in the rostromedial lobes, the valvula cerebelli (Va) and corpus cerebelli (CCe), form a layer that is deep to the molecular layer. These GCs are derived from neuronal progenitors located in the rostral part of the rhombic lip, which migrate ventrally. Each GC sends its axon to meet the dendrites of Purkinje cells (PCs) in the molecular layer. In contrast, the GCs in the caudolateral lobes, the eminentia granularis (EG) and lobus caudalis cerebelli (LCa), are derived from neuronal progenitors in the caudal and lateral parts of the rhombic lip, and their somata lie superficial to the molecular layer. They send their axons to PCs in the cerebellum, and also extend them caudally to the dendrites of crest cells, which are Purkinje-like cells, in the dorsal hindbrain. While rostromedial GC circuits involving PCs are likely to be involved in motor learning and classical conditioning, the caudolateral GC circuits, involving both PCs and the crest cells are may control motor coordination in response to vestibular information [12,13]. The mechanisms responsible for the formation of these two distinct GC circuits remain unknown.

The extracellular matrix (ECM) controls neural circuit formation in various ways [14]. Collagen proteins are widely expressed in the ECM of the developing nervous system and its surrounding tissues [15,16], and have been suggested to control axon extension and axon guidance in vertebrates and invertebrates [17–21]. Among the collagens, type IV collagen is a heterotrimeric protein complex, whose protomers can include various combinations of subunits (Col4a1-6); this complex is a component of the basement membrane (BM) that lines epithelial cell sheets [22]. In humans, type IV collagen gene mutations, e.g., *COL4A5*, lead to Alport syndrome [23], of which most symptoms, including renal dysfunction and auditory disturbance, are attributed to defects in the BM structure [22,23].

The zebrafish *col4a5* mutant, *dragnet*, shows abnormal targeting of the retinal ganglion cell (RGC) axons; in wild-type animals, individual retinal axons project to single layers in the optic tectum, while the axons in *dragnet* aberrantly pass between layers or terminate on multiple layers [24]. Because type IV collagen binds to the axonal guidance molecule Slit1 [25], one proposed explanation for the aberrant RGC axogenesis in these mutants is the presence of an abnormal Slit concentration gradient in the optic tectum [25]. In addition to regulating guidance molecules, collagens regulate neurite growth through integrin-family receptors or discoidin domain receptor 1 (DDR1) [26–29], and they control cell migration or tissue morphogenesis through adhesive G protein-coupled receptors (aGPCRs) [30,31]. However, it is still largely unknown how collagens control the axogenesis of individual types of neurons.

We previously isolated zebrafish mutants with defects in the formation of cerebellar neurons and their neurites [9]. Among them, *shiomaneki* (*sio)* larvae show aberrant axogenesis of the GCs in the caudolateral lobe. In this study, we identified *col4a6* as the causative gene for the *sio* mutation, and demonstrated that type IV collagen controls GC axon formation by regulating the integrity of the BM.

## Results

### Three Types of GCs Form Two Distinct Neural Circuits in the Zebrafish Cerebellum

First, to reveal the normal axonal structures of zebrafish GCs, we performed single-cell labeling of GCs using GC-specific Gal4 Tg lines, gSA2AzGFF152B and gSAIGFF23C [32]. We took two approaches for mosaic labeling: the use of a partially silenced UAS reporter line and the transposon Tol1-mediated incorporation of reporter DNA [32]. Larvae were obtained from a cross between the gSA2AzGFF152B line and the partially silenced UAS:Kaede reporter fish (Fig 1A-1C) or by injecting UAS:Kaede reporter DNA and Tol1 transposase RNA into cleavage-stage embryos of the gSAIGFF23C line [32]. Larvae expressing Kaede in one or a few GCs were selected at 5 days post fertilization (dpf), and the structure of the GCs in the larval cerebellum was examined (Fig 1A-1D).

We found that GCs in the Va and CCe (rostromedial lobes) extended their axon dorsally. It bifurcated in the superficial domain (molecular layer), and then extended bilaterally to the dendrites of PCs (parallel fibers, Fig 1A, 1B and 1Ea). This T-shaped axonal structure was similar to that of GCs in the mammalian cerebellum [4].

The GCs in the EG displayed a similar T-shaped axonal structure and sent their axonal branches ipsilaterally and contralaterally; however, both branches extended caudally to the dorsal hindbrain after reaching the lateral edge of the cerebellum (Fig 1Eb). The GCs in the LCa extended their axon only ipsilaterally; it then extended caudally to the dorsal hindbrain, like the GC axons from the EG (Fig 1Ec). Thus, the GCs from the EG and LCa (caudolateral lobe) extended a long axon that first synapsed with the dendrites of PCs in the cerebellum, and then with the dendrites of crest cells in the crista cerebellaris, which is part of the dorsal hindbrain (Fig 1E). These data are consistent with previous findings obtained from the retrograde labeling of the dendrites of crest cells, which indicated that the GCs in the LCa and EG project ipsilaterally and bilaterally to the crest cells, respectively [11].

Our results revealed that there are three types of GCs (Va/CCe, LCa, and EG); that the axonal structure of the GCs in the rostromedial lobes (Va/CCe) is different from those of the GCs in the LCa and EG; and that the axons of the caudolateral lobes (LCa and EG) extend caudally to the crest cells. Thus, the rostromedial and caudolateral GCs form two distinct neural circuits.

### Type IV Collagen Is Required for GC Axogenesis in the Caudolateral Lobes

**Fig 2:**
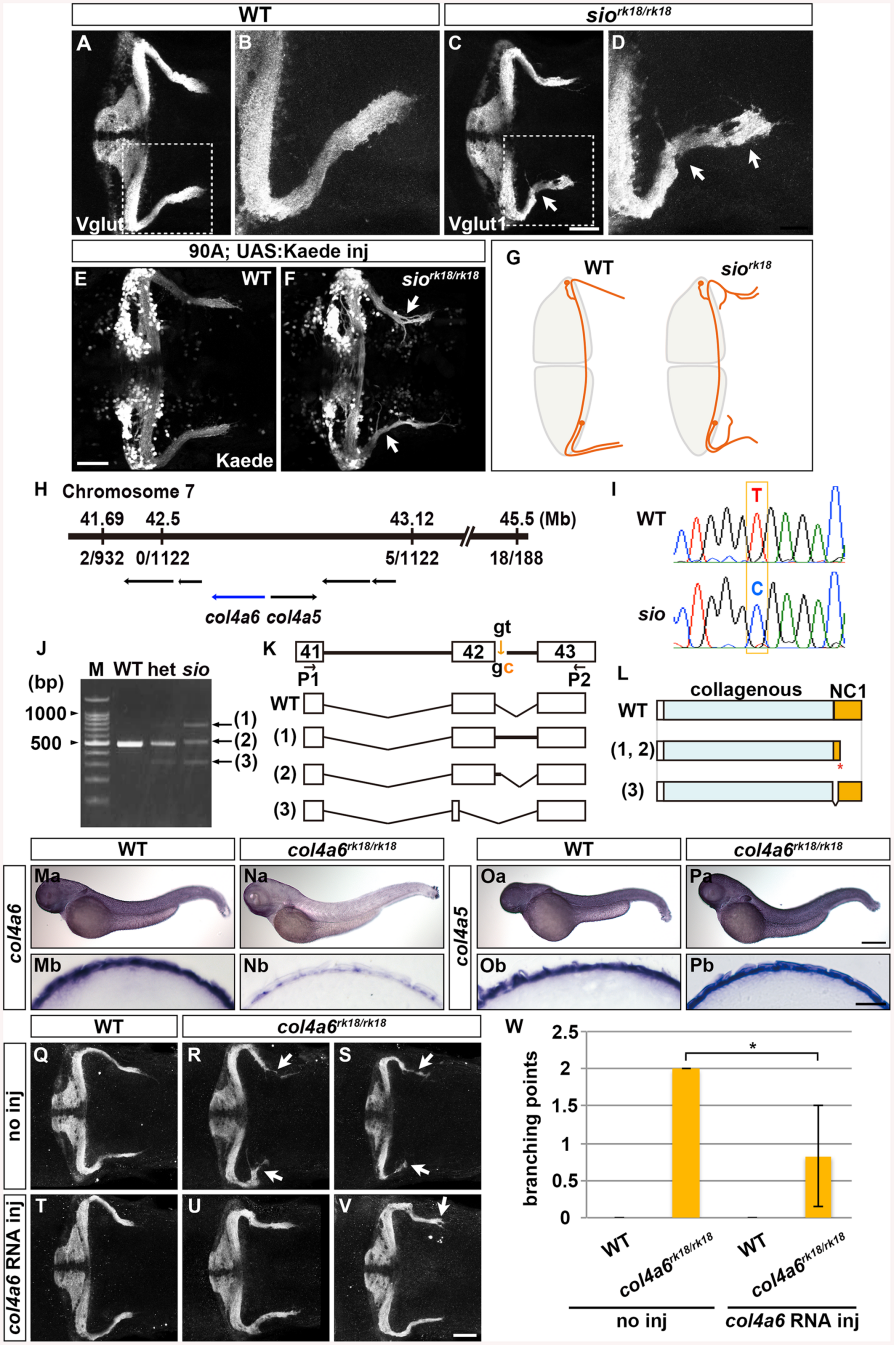
Type IV collagen gene *col4a6* is required for axogenesis of the GCs in caudolateral lobes. (A-D) Staining with an anti-Vglut1 antibody, which marks the presynaptic termini of GC axons, revealed that the GCs in the caudolateral lobes of 5-dpf homozygous *shiomaneki* (*sio* ^*rk18/rk18*^) mutants had abnormal axons, which formed abnormally branched bundles (C, D). Dorsal projection views of wild-type control (A, B) and *sio* mutant (C, D) cerebella. (E, F) Labeling of the caudolateral GCs in the GC-specific Gal4 line hspGFFDMC90A also showed aberrant axogenesis (marked by arrowheads in F). Dorsal views of the control (E) and *sio* mutant (F) cerebella. (G) Schematic representation of *sio* phenotypes. Dorsal views. (H) The *sio* locus was mapped to chromosome 7, between 41.69 and 43.12 Mb from the telomere. The type IV collagen genes *col4a6* and *col4a5* are located in this region. The numbers of recombinations in the mutant genomes are indicated. (I) The mutant contained a T-to-C point mutation in a splicing donor site of the *col4a6* gene. (J) Detection of mutant transcripts by RT-PCR with primers (P1, P2) shown in K. (K) Structures of the wild-type and mutant transcripts. (L) Structures of the wild-type and mutant proteins. ‘Collagenous’ and ‘NC1’ represent the collagenous domain and non-collagenous domain 1, respectively. The mutant transcripts and proteins in K and L correspond to the PCR bands indicated by the same number in J. (Ma-Pb) Expression of *col4a6* (Ma, Na) and *col4a5* (Oa, Pa) in wild-type (Ma, Mb, Oa, Ob) and *sio* (Na, Nb, Pa, Pb) larvae at 3 dpf. Lateral views (Ma-Pa) and cross sections of the dorsal hindbrain region (Mb-Pb). (Q-W) The abnormal axogenesis of the caudolateral GCs in the *sio* mutants was suppressed by expressing wild-type *col4a6*. *col4a6* RNA (50 pg) was injected into one-cell-stage embryos from a cross between *sio* heterozygotes (T-W). The resultant 5-dpf larvae were stained with an anti-Vglut1 antibody. Uninjected controls for wild-type (Q) and *sio* ^*rk18/rk18*^ (R, S) larvae are also shown. Some of the injected *sio* larvae showed normal GC axons (U), and others showed a relatively weak abnormality in the GC axons (V). The number of abnormal GC axon branches in a larva is indicated (W). The abnormal branch points were counted in the uninjected wild-type (4 larvae) and *sio* mutants (3 larvae) and in the *col4a6* RNA-injected wild-type (3 larvae) and *sio* mutants (6 larvae), and the means were calculated. The injection of *col4a6* RNA significantly reduced the number of abnormal branch points (*, P<0.05). Scale bars: 50 μm in A, C; 20 μm in B, D; 500 μm in Pa (applied to Ma, Na, Oa); 25 μm in Pb (applied to Mb, Nb, Ob); 50 μm in V (applied to Q, R, S, T, U).

To elucidate the molecular mechanisms controlling GC axon development, we investigated the zebrafish mutant *sio*, which shows abnormal GC axon projections to the crest cells [9]. In the mutant larvae, the Vglut1^+^ axons of the GCs in the caudolateral lobes were aberrantly branched or misoriented in the crista cerebellaris (*n* = 10/10) (Fig 2A-2D). Labeling of the GCs with GC-specific Gal4 lines confirmed that axogenesis was affected for the GCs in the caudolateral lobes (hspDMC90A, *n* = 4/4, Fig 2E and 2F; gSA2AzGFF152B, *n* = 2/2, S1 Fig). Sparse cell labeling with the GC-specific Gal4 lines further revealed that most of the GC axons from the LCa and EG displayed misoriented axon(s) (LCa: 100%, *n* = 3; EG: 73.3%, *n* = 15), whereas only a portion of the GC axons in the CCe showed aberrant axons in the mutant larvae (14.7%, *n* = 34, S2 Fig, the statistic analysis is shown in S1 Table). Expression of the differentiated GC marker Neurod was not affected in the *sio* mutants (S3 Fig), suggesting that the *sio* locus is involved in GC axogenesis but not in their differentiation (Fig 2G).

To reveal the molecular nature of the *sio* locus, we performed positional cloning, and mapped the locus to a region in chromosome 7 that contains the type IV collagen genes *col4a5* and *col4a6* (Fig 2H). Sequence analysis identified a T-to-C point mutation in the splicing donor on the 3’ side of the 42nd exon of the *col4a6* gene in the *sio* mutant genome (Fig 2I). Reverse Transcription (RT)-PCR revealed that three aberrant *col4a6* transcripts were generated by abnormal splicing in the *sio* mutants (Fig 2J and 2K). Two of the aberrant transcripts encoded a C-terminally truncated Col4a6, and one encoded a mutant Col4a6 containing an internal deletion (Fig 2L). All of the mutant proteins had a deletion in the Noncollagenous Domain (NC1), which is required for the assembly of type IV collagen [33].

Col4a6 forms a heterotrimeric complex with Col4a5 [22], and *col4a5* is expressed in the epidermal cells surrounding the tectum in zebrafish [24]. We therefore examined the expression of *col4a5* and *col4a6* in the *sio* mutant hindbrain region. In wild type, both *col4a5* and *col4a6* transcripts were detected in the epidermal cells surrounding the hindbrain (Fig 2Ma, 2Mb, 2Oa and 2Ob). Although the expression of *col4a5* was not affected in the *sio* mutants (Fig 2Pa and 2Pb), the expression of *col4a6* was prominently reduced (Fig 2Na and 2Nb), suggesting that the *col4a6* transcripts in the mutants underwent nonsense-mediated mRNA decay. The injection of *col4a6* RNA suppressed the formation of aberrant axons of the GCs in the *sio* mutants (Fig 2Q-2W). In this experiment, exogenous *col4a6* was provided ubiquitously, but Col4a6 may function only in epidermal cells in which its heteromeric partner, Col4a5, is present. These data collectively indicated that the *sio* locus encodes Col4a6 (hereafter we refer to the *sio* mutant as the *col4a6* mutant).

**Fig 3:**
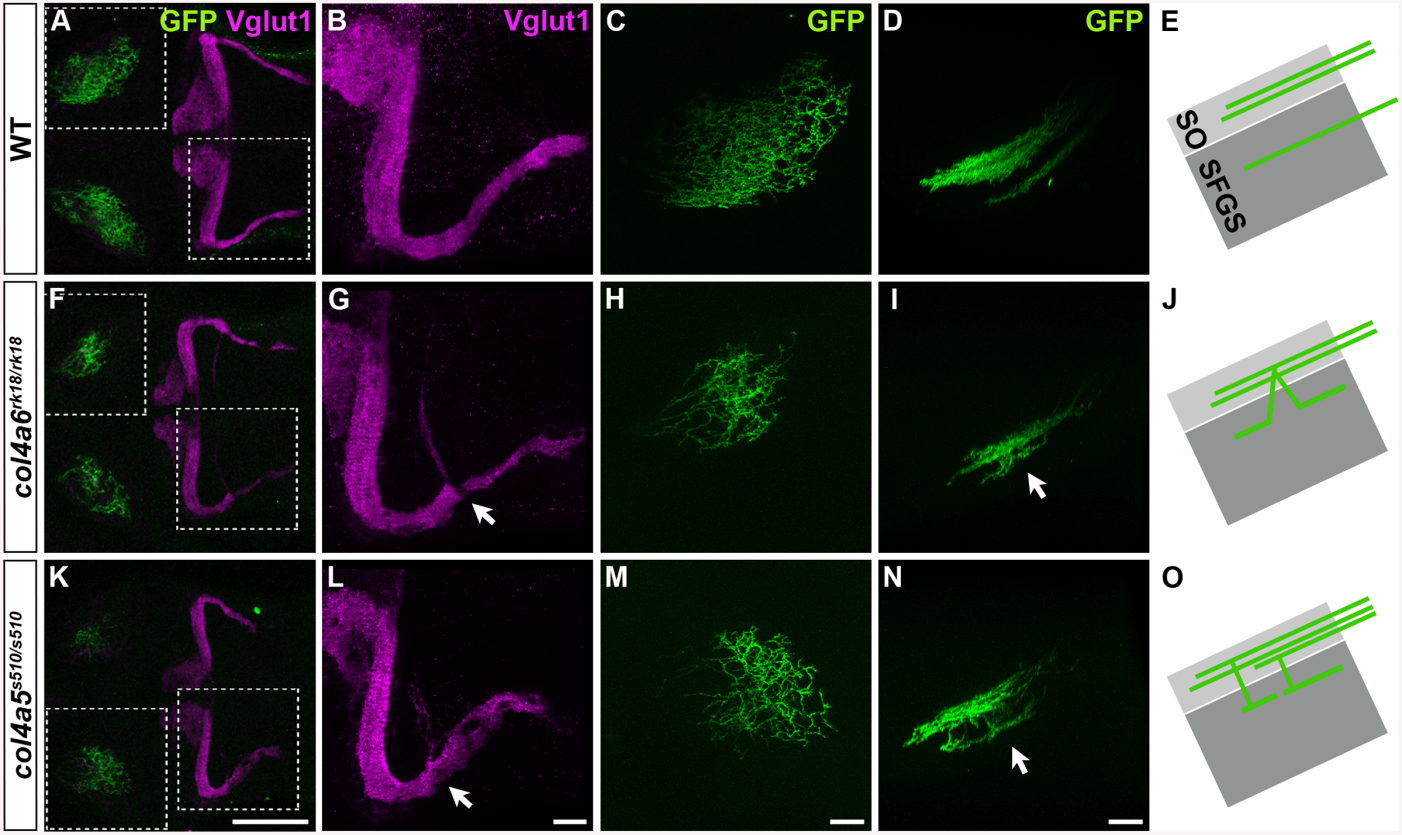
The Col4a5 and Col4a6 complex plays a role in the axogenesis of GCs and RGCs. Wild-type (A-E), *col4a6* ^*rk18/rk18*^ (F-J), and *col4a5* ^*s510/s510*^ (K-O) mutant 5-dpf larvae harboring the pou4f3:Gal4, UAS:GAP-GFP transgene, which labels the axons of retinal ganglion cells (RGCs) in a mosaic manner, were stained with anti-Vglut1 (magenta) and anti-GFP (green) antibodies. Dorsal projection views (A-C, F-H, K-M) of the midbrain/hindbrain (A, F, K), left half of the rostral hindbrain (B, G, L), and half of the tectum (C, H, M). Lateral view of the tectum (D, I, N). Both *col4a6* and *col4a5* mutant larvae showed abnormal branching of the GC bundles (indicated by arrows, G, L). In wild type animals, each RGC projected to a single layer in the tectum (D), whereas in the *col4a6* and *col4a5* mutants some RGC axons trespassed between tectal layers (marked by arrows in I and N). (E, J, O) Schematic drawing of the RGC axons (lateral view). SO, stratum opticus; SFGS, stratum fibrosum et griseum superficiale. The statistic analysis is shown in S2 Table. Scale bars: 100 μm in K (applied to A, F); 20 μm in L (applied to B, G); 20 μm in M (applied to C, H); 20 μm in N (applied to D, I).

Zebrafish *col4a5* mutants are reported to exhibit abnormal axon targeting of RGCs to the tectum [34]. We therefore examined the GC and RGC phenotypes in the *col4a5* and *col4a6* (*sio*) mutants (Fig 3). Mosaic labeling of RGCs with the pou4f3:Gal4, UAS:GAP-GFP reporter line showed that some of the RGC axons trespassed between tectal layers in the *col4a6* mutant larvae (*n* = 4/4) (Fig 3H and 3I), as in the *col4a5* mutants [24] (*n* = 3/3) (Fig 3M and 3N). Similarly, we detected aberrant Vglut1^+^ GC axons in the caudolateral lobes of the cerebellum in both the *col4a5* (*n* = 3/3) (Fig 3K and 3L) and *col4a6* mutants (*n* = 5/5) (Fig 3F and 3G). Expressivity and penetrance of the aberrant RGC and GC axons were not significantly different between *col4a6* and *col4a5* mutant larvae (S2 Table). These data indicate that both Col4a5 and Col4a6 are required for the proper axogenesis of RGCs and caudolateral GCs, and imply that the Col4a5/Col4a6 complex plays a role in the axogenesis of these neurons in zebrafish.

### Slit May Not Be Involved in GC Axogenesis

**Fig 4:**
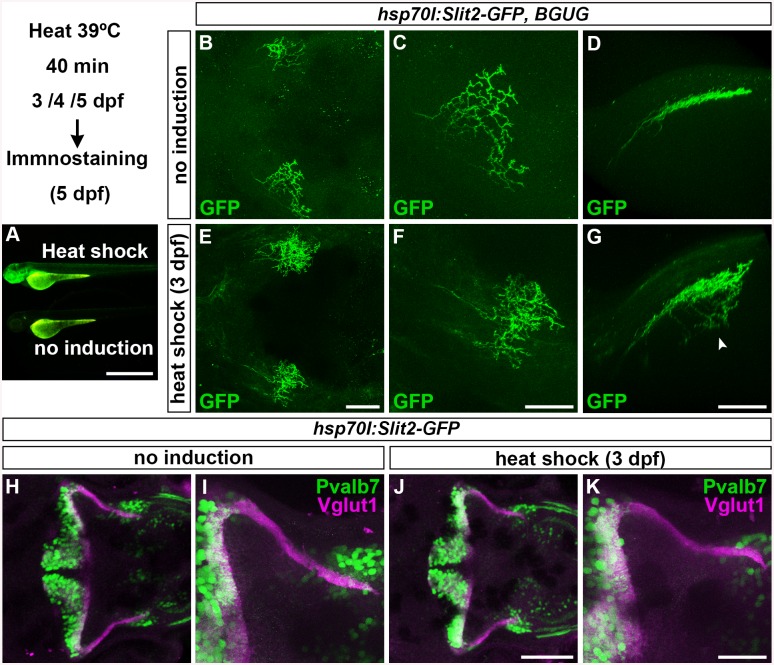
Slit may not be involved in the axogenesis of GCs. Slit2 was overexpressed using the hsp70l:Slit2-GFP; pou4f3:Gal4 UAS:GAP43-GFP (B-G), or hsp70l:Slit2-GFP (H-K) lines during development of the RGC and GC axons (3/4/5 dpf). The structure of the RGC and GC axons was examined at 5 dpf. Slit2-GFP was detected after 40 min of heat shock (A, lower panel). (B-G) Heat shock-induced overexpression of Slit2-GFP at 3 dpf induced abnormal axonal projections (trespass) of the RGCs (*n* = 2/2, indicated by an arrowhead in G). The RGC axons were visualized by immunohistochemistry with an anti-GFP antibody. Control non-induced (B, C, D) and heat-shocked larvae (E, F, G). Low- (B, E) and high-magnification dorsal views (C, F), and lateral views (D, G) of the tectum. (H-K) Heat shock-mediated overexpression of Slit2-GFP at 3 dpf did not affect the formation of GC axons (*n* = 10/10, J, K). The larvae were stained with anti-Vglut1 (GC axons, magenta) and parvalbumin7 (Pvalb7, PCs, green) antibodies. Overexpression at 4 or 5 dpf also did not affect the GC axons (S4 Fig). Statistic analysis is shown in S3 Table. Scale bars: 1 mm in A; 50 μm in E (applied to B), 40 μm in F (applied to C); 40 μm in G (applied to D); 100 μm in J (applied to H); 40 μm in K (applied to I).

We next examined how type IV collagen controls RGC and GC axogenesis. Type IV collagen proteins are generated in epidermal cells and deposited into the BM underlying the epidermal cells. Type IV collagen is proposed to regulate the gradient of the axon guidance molecule Slit1 [24]. Neither the *slit* nor the *robo* genes, which encode Slit receptors, are strongly expressed in the antero-dorsal hindbrain, with the exception of *robo3*, which is expressed in the cerebellum and thought to function as a negative regulator of Slit signaling [35] (S4 Fig). We therefore examined whether overexpressing Slit protein would affect the formation of GC axons, by using the transgenic line hsp70l:Slit2-GFP [36] (Fig 4A), which was previously used to analyze the Slit-mediated axon targeting of RGCs [24]. As reported previously [24], the overexpression of Slit2-GFP induced aberrant RGC axons (*n* = 2/2, Fig 4E-4G). However, overexpressing Slit2-GFP at 3, 4, or 5 dpf, when the GC axons extend to the crest cells, did not significantly affect the GC axogenesis (for 3 dpf, *n* = 1/10, Fig 4J and 4K; for 4 dpf, *n* = 0/6, S5C and S5D Fig; for 5dpf, *n* = 0/5, S5E and S5F Fig; the statistic analysis is shown in S3 Table). These data suggest that Slit signaling is not involved in the axogenesis of caudolateral GCs, and thus that type IV collagen regulates the axogenesis of caudolateral GCs by a mechanism other than controlling Slit proteins.

### Dorsal Hindbrain BM Structure Is Disrupted in Type IV Collagen Mutants

**Fig 5:**
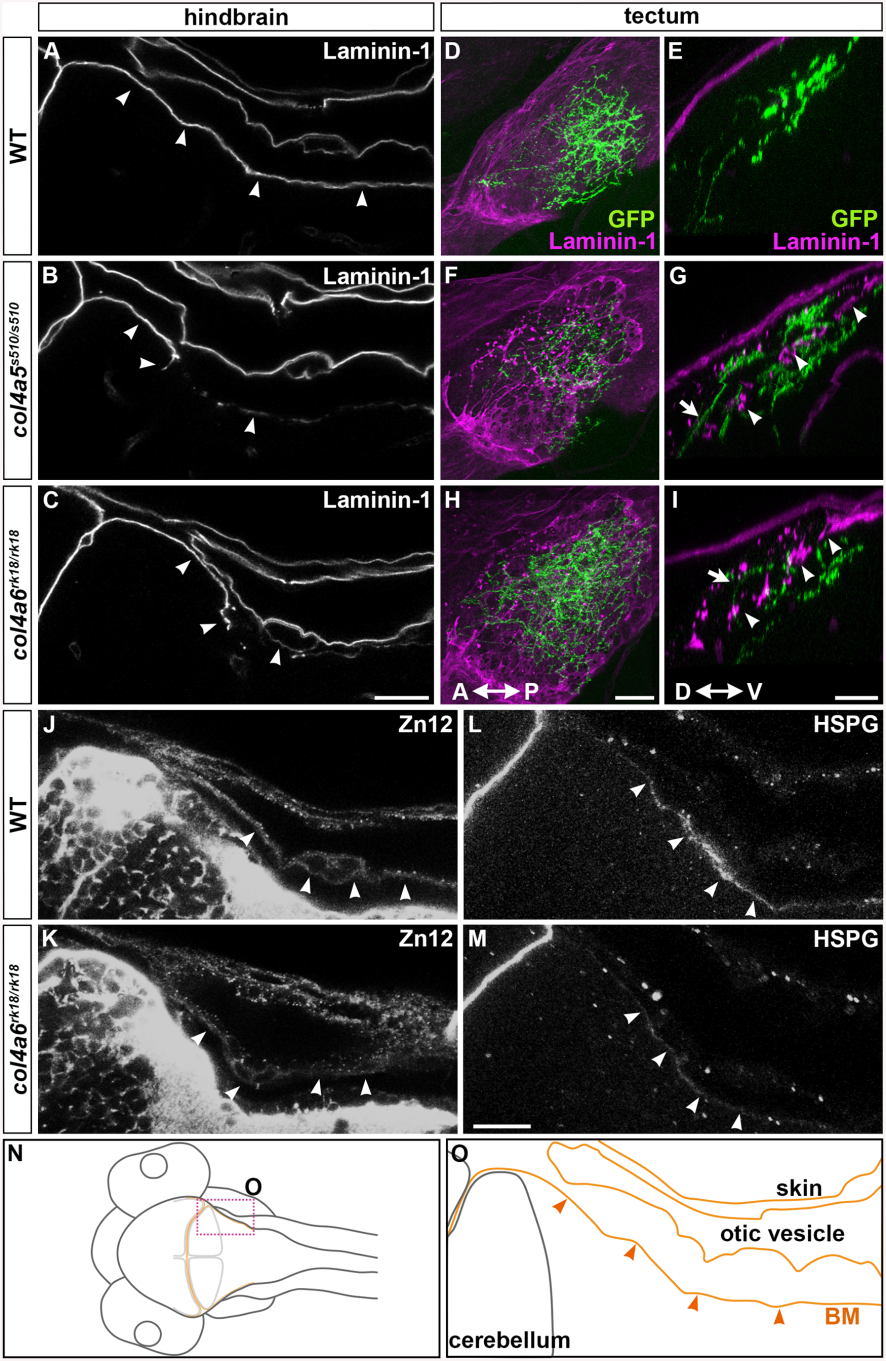
Col4a6 and Col4a5 are required for BM integrity. Wild-type (A, D, E, J, L) and *col4a5* (B, F, G) and *col4a6* (C, H, I, K, M) mutant larvae at 5 dpf were stained with anti-laminin–1 (A-I), anti-HNK–1 (zn12, J-K), or anti-HSPGs (10E4, L, M) antibodies. (A-C, J-M) BM in the dorsal hindbrain. Dorsal views of the rostral hindbrain, optical sections. (D-I) BM in the tectal region. RGC axons marked by pou4f3:Gal4; UAS:GAP-GFP (green) were co-stained with an anti-GFP antibody in the tectum; dorsal projection views (D, F, H) and lateral views (E, G, I). The laminin–1^+^ BM structure was split in the dorsal hindbrain region of the *col4a5* and *col4a6* mutants (indicated by arrowheads, B, C). In the tectal region of these mutants, the laminin–1^+^ BM was split into two layers: one attached to the skin and the other located more internally with intermittent disruption (arrowheads, G, I). Some RGC axons were located in the interspace between the two BMs in the mutant tectum (indicated by arrows, G, I). Signals for HNK–1 and HSPGs (marked by arrowheads) in the dorsal hindbrain BM of the *col4a6* mutants (K, M) were weaker than in wild type (J, L). (N, O) Schematic drawing of head (N) and hindbrain (O) regions. The laminin–1^+^ structures are marked by brown lines and the BM surrounding the hindbrain is marked by arrowheads (O). The statistic analysis is shown in S4 Table. Scale bars: 20 μm in C (applied to A, B); 20 μm in H (applied to D, F); 30 μm in I (applied to E, G); 20 μm in M (applied to J, K, L, M).

A triple helix complex of Col4a5 and Col4a6 exists in the BM of human skin, smooth muscle, and kidney, and is required for proper BM formation [22]. We therefore examined the structure of the BM surrounding the dorsal hindbrain and optic tectum of the *col4a5* and *col4a6* mutants (Fig 5). Immunostaining with an anti-laminin–1 antibody revealed a linear structure of the BM in the tectum and hindbrain of 5-dpf wild-type larvae (Fig 5A, 5D and 5E). Although the BM initially adheres directly to the skin cells (Fig 2Mb and 2Ob), the BM was separated from the skin by the otic vesicles that grew rapidly at the early larval stage (Fig 5A, 5N and 5O). In the dorsal hindbrain of *col4a5* and *col4a6* mutants, the laminin–1 signals were split or intermittently disrupted (Fig 5B and 5C, S4 Table). In the tectal BM of these mutants, the laminin-1-positive BM was split into two layers: one attached to the skin and the other more internal, with intermittent disruption (arrowheads, Fig 5G and 5I; S4 Table). Some RGC axons were observed in the interspace of the split BM structures (arrows, Fig 5G and 5I).

We also examined the distributions of two tectal BM components, zn12 (HNK–1) glyco-epitope and heparan sulfate proteoglycans (HSPGs), in the *col4a6* mutants, because their distributions are altered in *col4a5* mutants [24]. We found that compared to wild type (Fig 5J and 5L), the HNK–1 epitope and HSPGs at and near the hindbrain BM were more sparse in the *col4a6* mutants (Fig 5K and 5M, S4 Table). The HNK–1^+^ (zn12^+^) region was also thicker in the tectal BM region of the *col4a6* mutants (S6 Fig, S4 Table).

**Fig 6:**
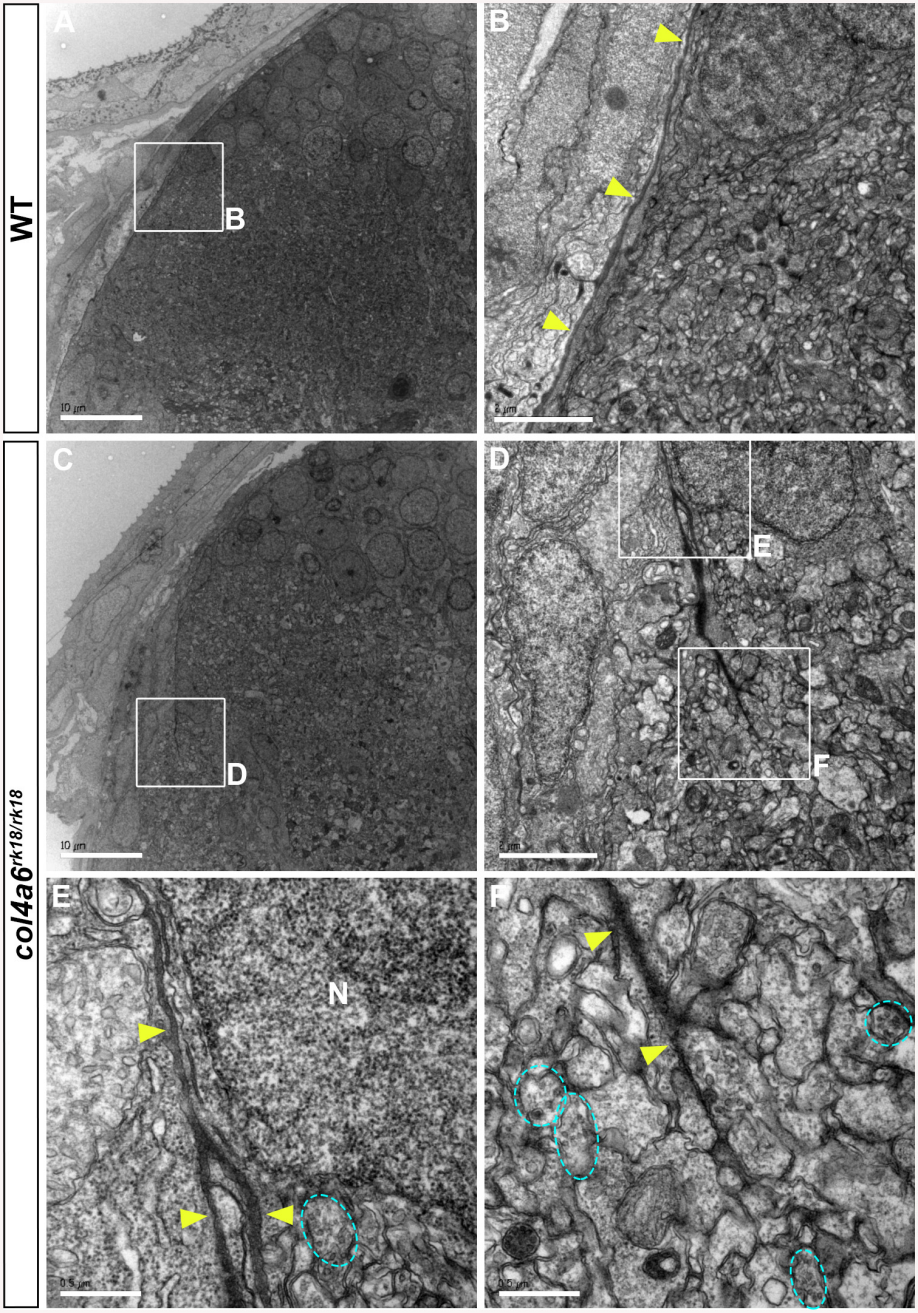
BM structure is disrupted in the *col4a6* mutant hindbrain. Dorsal hindbrain of 5 dpf wild-type (A, B) and *col4a6* mutant (C-F) larvae was analyzed by electron microscopy. Cross sections (A, C). (B, D) Higher-magnification views of box B in A and box D in C. (E, F) Higher-magnification images of boxes E and F in D. The BM is indicated by yellow arrowheads. Axons containing synaptic vesicles are marked by blue dashed circles. The BM was branched (E) or truncated (F) in the *col4a6* mutant hindbrain. N, nucleus. Scale bars: 10 μm in A; 2 μm in B; 10 μm in C; 2 μm in D; 0.5 μm in E; 0.5 μm in F.

Electron microscopic (EM) analysis in wild-type larvae showed intact BM surrounding the tectum and dorsal hindbrain, where the GC axons run (Fig 6A and 6B). In contrast, the electro-dense BM structure in the *col4a6* mutant hindbrain contained branched and/or truncated regions (Fig 6C-6F). Some axons that could be distinguished by the presence of presynaptic vesicles were observed outside the BM (Fig 6F). Truncation of the tectal BM and ectopic localization of the RGC axons were also detected in both *col4a5* and *col4a6* mutants by the EM analysis (S7 Fig). These data collectively indicated that type IV collagen is required for the integrity of both the tectal and dorsal hindbrain BM.

### Abnormal GC Axon Formation and Regeneration Is Coupled with Abnormal BM Structure

**Fig 7:**
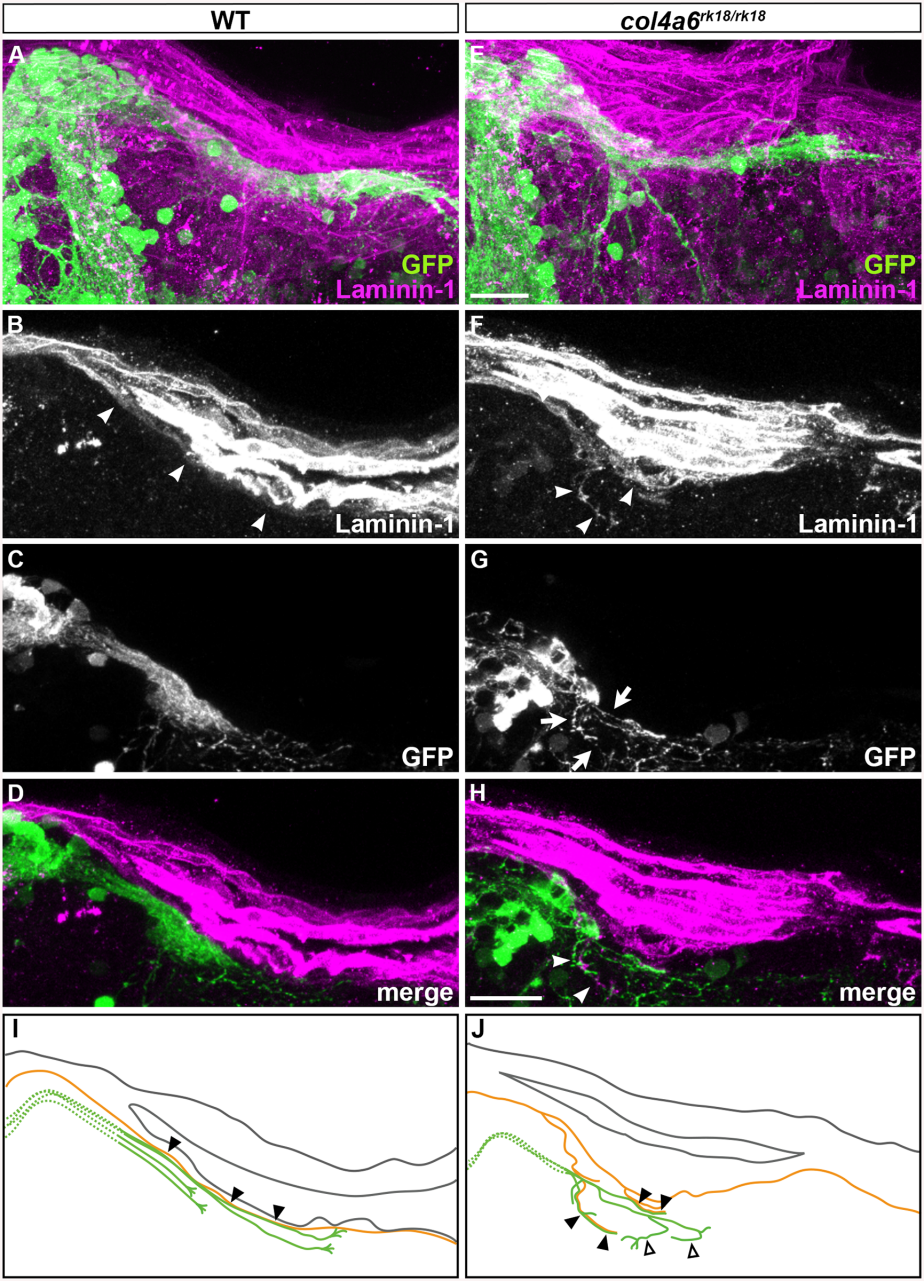
Abnormal BM structure is coupled with the abnormal axogenesis of GCs in *col4a6* mutants. Wild-type (A-D) and *col4a6* mutant (E-H) transgenic larvae that express GFP in the GC (hspGFFDMC90A; UAS:GFP) were stained at 5 dpf with anti-GFP (green) and anti-laminin–1 (magenta) antibodies. Dorsal projection views (A, E); 9.723-μm-thick projection views (B-D, F-H). (I, J) Schematic representation of BM (brown) and GC axons (green) in wild-type (A-D) and the *col4a6* mutant (E-H) hindbrain. The laminin–1^+^ BM structure was split into two layers in the *col4a6* mutant (indicated by arrows in G). In the same region, the corresponding GC axons were bifurcated, some of the axons ran along the split BM layers (indicated by arrowheads in F and H, by closed triangles in J) and some of them did not run along the BM layer (indicated by open triangles in J). The root of the GC axons cannot be distinguished from one another and thus is described by a dotted line. Scale bars: 20 μm in E (applied to A); 20 μm in H (applied to B, C, D, F, G).

To address the role of the BM in GC axogenesis, we carefully examined the relationship between the BM and caudolateral GC axons in the wild-type and *col4a6* mutant hindbrain. The GC axons were labeled with the GC-specific Gal4 line hspGFFDMC90A and UAS:GFP [32], and the BM was stained with the anti-laminin–1 antibody in the wild-type and *col4a6* mutant dorsal hindbrain (Fig 7). The GFP^+^ axons of the caudolateral GCs ran along the linear BM in the wild-type hindbrain (Fig 7A-7D and 7I).

In contrast, in the *col4a6* mutant, the BM split into two layers in the dorsal hindbrain (Fig 7F and S8 Fig), and the GC axon bundle bifurcated at the split (Fig 7F-7H and S8 Fig). After the bifurcation, some of the GC axons ran along the abnormal BM layer (indicated by closed triangles, Fig 7 and S8 Fig) but some of them did not run along the BM (open triangles, Fig 7 and S8 Fig). Corresponding splits of the BM and the GC axon bundles were observed in all of the homozygous *col4a6* mutant larvae observed (5 mutant larvae had 10 branches of the GC axon bundles; all of them were coupled with the BM splits), suggesting that the BM splits caused the GC axons to branch.

**Fig 8:**
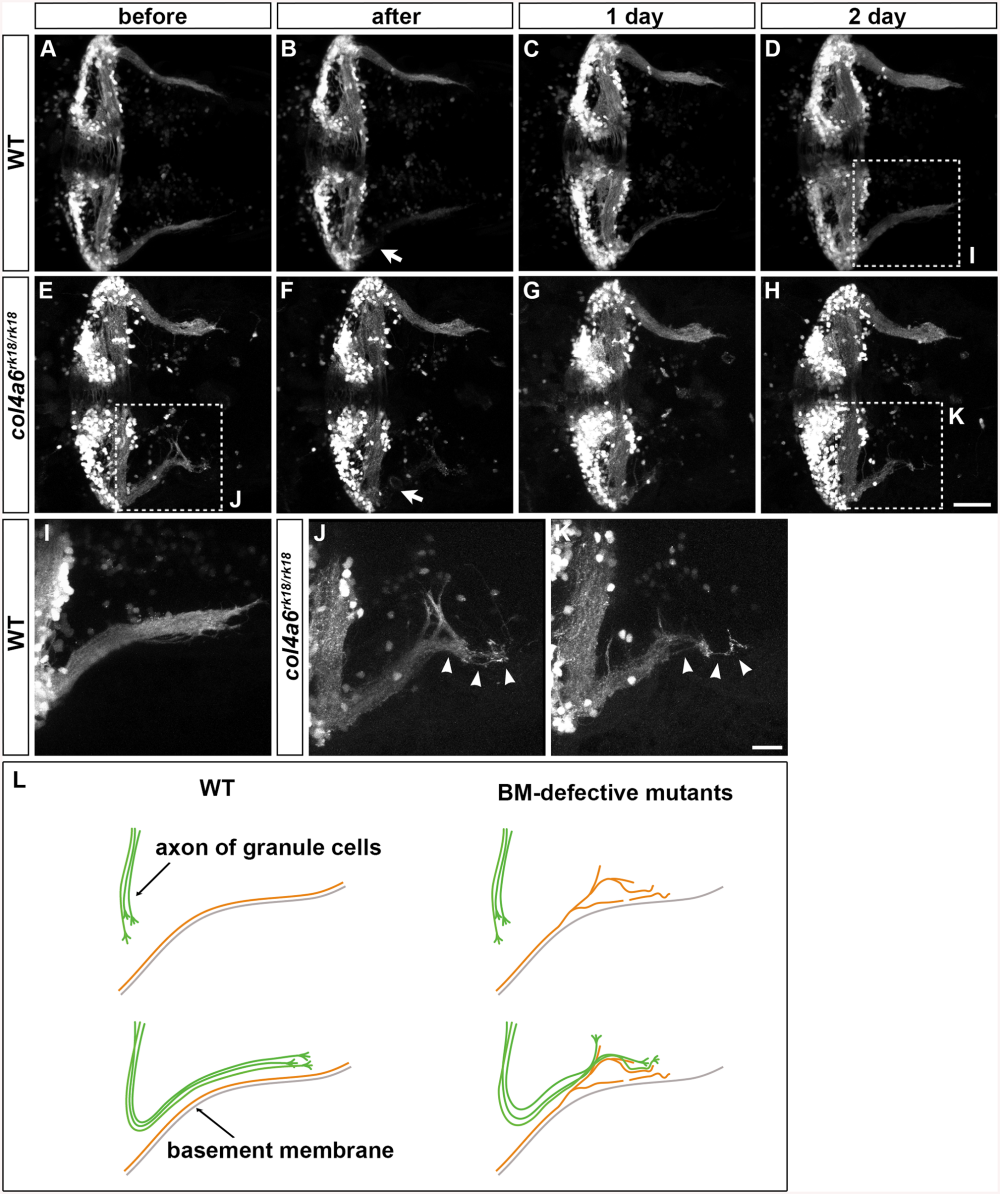
Abnormality in regenerating GC axons is linked to the abnormal BM structure in *col4a6* mutants. The axons of caudolateral GCs in wild-type and *col4a6* mutant larvae harboring the hspGFFDMC90A (GC-specific Gal4) and UAS:GFP transgenes were ablated by a laser on the left side (marked by arrows) at 5 dpf. The GC axons of the larvae were observed before (A, E), soon after (B, F), or 1 (C, G) or 2 days (D, H) after the laser ablation. Dorsal views of the rostral hindbrain regions. (I, J, K) High-magnification images of boxes I (in D), J (in E), and K (in H). Some of the regenerated axons followed the same abnormal routes that were used by the original axons in the *col4a6* mutant (indicated by arrowheads in J and K). (L) Schematic representation of the axogenesis of caudolateral GCs in wild-type and BM mutant (*col4a5* and *col4a6*) larvae. Scale bars: 50 μm in H (applied to A-G); 20 μm in K (applied to I, J). (See more examples in S5 Fig).

We next ablated the axon bundles of the caudolateral GCs extending to the crest cells in 5-dpf wild-type larvae, by using a two-photon laser, and observed their regrowth. The re-growing axon bundles potentially contained both regenerated and de novo generated GC axons (Fig 8C and S9 Fig). After two days, these axons had reconstructed the neural circuits to the crest cells (Fig 8D and S9 Fig). We then performed this experiment using the *col4a6* mutant. If the BM abnormality led to the aberrant GC axogenesis observed in this mutant, the newly formed (and possibly regenerated) axons should follow abnormal routes similar to those used by the original GC axons in the BM-affected areas.

Following axon ablation in the *col4a6* mutants, the caudolateral GC axons were re-extended as in wild type, although the extended axons were abnormal (Fig 8E-8H and S9 Fig). Notably, at least some of the newly formed axons followed abnormal routes that were similar or identical to those used by the original axons (marked by arrowheads in Fig 8J, 8K and S9 Fig). These findings suggest that the abnormal BM structure is directly linked to the abnormal GC axogenesis in the *col4a6* mutant.

## Discussion

### Three types of GCs in zebrafish cerebellum

The GCs in zebrafish are known to form two different neural circuits. The GCs in the rostromedial lobes have a T-shaped axon that projects to the dendrites of PCs, whereas the GCs in the caudolateral lobes send their axons to PCs in the cerebellum and to crest cells in the dorsal hindbrain [9–11]. By mosaic analysis using GC-specific Gal4 lines, we confirmed these circuits and further revealed that, within the caudolateral lobes, the GCs in the EG and LCa exhibit different axonal structures: the GCs in the EG have a T-shaped axon that projects contralaterally and ipsilaterally to the crest cells, whereas the GCs in the LCa send their axon only ipsilaterally to the crest cells (Fig 1). The developmental processes of the GCs in the three lobes are also different [10,11], and some development-related genes are differentially expressed between the GCs of the rostromedial and caudolateral lobes [9], suggesting that distinct molecular mechanisms control the axogenesis of the three types of GCs. In this report, we found that the axons of the GCs in the caudolateral lobes were affected more strongly than those in the rostromedial lobes in *col4a5* and *col4a6* mutants (Figs 2, 3 and S2, S1 Table), suggesting that Col4a5 and Col4a6 differentially control GC axogenesis in a cell-population-specific manner. The parvalbumin7-expressing crest cells in the medial octavolateral nucleus, which are axonal targets of the caudolateral GCs, were not significantly affected (S10 Fig), indicating that type IV collagen controls the axogenesis of the GCs but not differentiation of their targets.

Signaling from type IV collagen in the pial layer to the collagen receptor DDR1 on GCs is reported to be involved in GC axon extension in mouse [26], implying that type IV collagen has a conserved role in GC axogenesis. The GC axons in mouse cerebellum display a T-shaped structure resembling that of the GCs in the rostromedial lobes of zebrafish cerebellum [4–7]. If the same signaling was used in mouse and zebrafish, the axons of the rostromedial GCs would be affected in type IV collagen zebrafish mutants. However only a small portion of the rostromedial GC axons were affected in *col4a6* mutants (Figs 2, 3 and S2, S1 Table). Furthermore, no *ddr1* expression was detected in zebrafish GCs (S11 Fig), indicating that collagen-DDR signaling may not be involved in zebrafish GC axogenesis. Nevertheless, Col4a5 and Col4a6 appear to be involved in the axogenesis of the caudolateral GCs in zebrafish.

### The Col4a5 and Col4a6 Complex Is Required for the Axogenesis of RGCs and GCs

In the human genome, the *COL4A5* and *COL4A6* genes are located side by side on the X chromosome [22]. Two COL4A5 molecules and one COL4A6 form a heterotrimeric complex through their NC1 domain, and the complex is deposited into the BM [22]. Similarly, the zebrafish *col4a5* and *col4a6* genes are located side by side on chromosome 7 (Fig 2), indicating a conserved synteny of these genes in vertebrates. Both *col4a5* and *col4a6* are expressed in epidermal cells (Fig 2), and a mutation in either gene resulted in similar abnormalities of the RGC and caudolateral GC axogenesis, as well as in the structure of the BM surrounding the tectum and hindbrain (Figs 3 and 5). Thus, a loss of Col4a6 cannot be compensated for by Col4a5, and vice versa, and both proteins have essential roles in RGC/GC axogenesis and BM integrity. Our data are consistent with the idea that Col4a5 and Col4a6 form a heterotrimeric complex in zebrafish, as they do in mammals. In humans, defects in the COL4A3/COL4A4/COL4A5 complex are the major cause of Alport syndrome, which is associated with a loss of BM integrity. Mutations in *COL4A6* alone have not been related to Alport syndrome [22,23], but a *COL4A6* mutation is linked to hereditary hearing loss, which is a more restricted symptom compared to Alport syndrome [37], implying that the Col4a5/Col4a6 complex has a more limited role in BM integrity in humans than in zebrafish.

There are three possible mechanisms by which type IV collagen controls axon outgrowth and pathfinding [14,17–21]. First, collagens in the BM bind to collagen receptors on neurons and induce neurite outgrowth. The major receptors for type IV collagen are integrin-family transmembrane proteins, such as α1β1 and α2β1 [27–29], and the binding of collagen to integrins initiates cytoplasmic signaling that activates focal adhesion kinase (FAK) [14]. Consistent with this mechanism, activated (phosphorylated) FAK was detected in the axons of the caudolateral GCs in wild-type larvae (S12 Fig). However, it was not significantly altered in the *col4a6* mutants (S12 Fig). Furthermore, blocking FAK activation with a chemical inhibitor did not affect GC axogenesis (S13 Fig). These data indicate that the Col4a5/Col4a6 complex may not initiate integrin signaling to activate FAK. Similarly, the morpholino-mediated knockdown of β1 integrin does not affect the axon targeting of RGC axons to the tectum [24]. Our data do not exclude the possibility that type IV collagen controls other aspects of neural development through unconventional collagen receptors, such as GPR56 and GPR126, which are reported to function in cell migration and/or tissue morphogenesis [30,31] or the possibility that the BM components other than the Col4a5/Col4a6 complex play roles in the GC axogenesis.

The second mechanism is that type IV collagen binds to guidance molecules and controls their concentration in and near the BM. Slit proteins are possible candidates, since type IV collagen binds to Slit1, and Slit overexpression or a mutation in the Slit receptor gene, *robo2*, affects the axon targeting of RGCs to the tectal layers [25]. However, overexpressing Slit did not affect the axogenesis of caudolateral GCs (Fig 4), suggesting that the Slit-Robo system is not involved in this axogenesis and that type IV collagen does not control GC axons by regulating the Slit gradient. Therefore, type IV collagen might have a different role in the axogenesis of RGCs versus GCs. However, we cannot exclude the possibility that type IV collagen controls the concentration gradient of guidance molecules other than Slits, and we note that still other mechanisms may be involved in the type IV collagen-mediated RGC axogenesis.

The third mechanism is supported by our findings, i.e., that type IV collagen controls the axogenesis of both RGCs and GCs by regulating the integrity of the BM, as discussed below, although we cannot rule out a role for guidance molecules or other mechanisms in this process.

### Role of Type IV Collagen in BM Integrity and in Axogenesis

Type IV collagen proteins in the BM are thought to confer tensile strength to the BM. We observed abnormalities in both the tectal and dorsal hindbrain BM of the *col4a5* and *col4a6* mutants, as revealed by laminin–1 staining, including branching, truncation, and thinning (Fig 5). The abnormal BM structure was further confirmed by EM analysis (Fig 6 and S7 Fig). Our data indicate that the Col4a5/Col4a6 complex is required for the integrity of the BM. In addition to the visible BM abnormalities, the distributions of the ECM components HNK–1 glyco-epitope and HSPGs in the vicinity of the BM were affected in the *col4a6* mutant hindbrain and tectum (Fig 5 and S6 Fig). Abnormal HNK–1 and HSPG distributions were reported for the *col4a5* mutants [24], supporting a role for the Col4a5/Col4a6 complex in the deposition of ECM components into the BM.

In a previous report, *col4a5* mutants did not show clear abnormalities in the laminin–1^+^ BM structure in the tectum [24]. The reason for the difference between our observations and the previous data on BM structures is unclear. One possibility is that, as the BM abnormalities did not always occur in the same positions and the BM lesions were discontinuous in the type IV collagen mutants, they might have been missed by other researchers. Even in the absence of the Col4a5/Col4a6 complex, the BM structure was not entirely abrogated, because other collagens and ECM components were present. Consistent with our observations of the BM, the abnormal branching and misorientation of the caudolateral GC axons were observed in different positions in each *col4a6* mutant larva (and also differed on the left and right sides, see Figs 2, 7 and 8). Similarly, the *col4a5* and *col4a6* mutants showed variations in the abnormal RGC axon targeting (Fig 3) [24].

It is possible that the Col4a5/Col4a6-defective BM is vulnerable to tensile force, and that its structure is disrupted by a physical force that accompanies larval growth. In any case, our data suggest that the BM abnormalities take place at relatively random positions, and that the abnormal BM structure led to abnormal axogenesis of the caudolateral GCs and possibly RGCs in the type IV collagen mutants.

### Role of the BM in the Formation of GC and RGC Axons

We found that the defective BM caused two types of aberrant axogenesis: (1) the presence of axons outside the nervous system, and (2) the branching and misorientation of axon bundles. In regions of BM disruption, some RGC axons wandered out of the tectum in *col4a5* and *col4a6* mutants, and some GC axons were observed outside the hindbrain in *col4a6* mutants (Figs 5 and 6). This situation is similar to that of the nephrons in Alport syndrome patients, in which blood cells escape from blood capillaries to the Bowman’s capsule through the disrupted BM [38].

Branched and misoriented GC axon bundles were observed in both type IV collagen mutants (Figs 2 and 7). Importantly, we found a close correlation between the regions of BM disruption and GC branching in the *col4a6* mutants. The axons of the caudolateral GCs ran along the BM in the hindbrain. At the branch points of the BM in the type IV collagen mutants, the axon bundles also branched, and many of the axon bundles ran along the abnormal BM (Fig 7), suggesting that the aberrant axons were guided by the abnormal BM structure. Furthermore, laser ablation of the GC axons revealed that the re-growing axons followed similar (or the same) abnormal routes as were used by the original GC axons in the *col4a6* mutants (Fig 8), suggesting that the normal axon path was broken in the mutants, preventing the axons from following the correct path to their target. The BM may serve to guide these axons to their target. Some of the abnormal axon bundles did not run along the BM after the branching in the *col4a6* mutant hindbrain (Fig 7J and S8 Fig), suggesting that the BM breaks caused misorientation of the caudolateral GC axons but did not attract them in some cases. The data support the idea that the BM integrity is essential for the axogenesis of the caudolateral GCs. A similar role of BM integrity is also reported for the mouse cerebellum, in which a deficiency of laminin α1, a subunit of laminin–1, results in corresponding defects in BM integrity and cerebellum development [39], suggesting that BM has a conserved role in cerebellum development.

The question remains, if the abnormal axogenesis was attributable to abnormal BM structures. Why were only the axons of the RGCs and GCs affected in the BM-defective mutants? An explanation may lie in the neuroanatomy of the developing cerebellar neural circuits. During the embryonic and larval stages, the major neural tracts run ventrally and are not located in the vicinity of the BM. The axons of the RGCs and GCs, however, form major axon tracts that are located most dorsally and are at least partially attached to BM. Therefore, the BM structure could play a pivotal and specific role in the axogenesis of these neurons.

To reach their correct targets, axons require proper guidance signaling. In the *col4a6* mutants, some GC axons still ran along the abnormal BM (Figs 7 and 8), suggesting that the BM itself, or a BM-associated molecule, functions to attract the axons. It is also likely that guidance molecules act on the GC axons independently of the BM. In any case, the BM and guidance molecules would need to cooperate to control the pathfinding of the GC axons. Eph-ephrin signaling plays a major role in guiding RGC axons [40,41]. More study is needed to clarify the signaling system that cooperates with the BM to guide the caudolateral GC axons.

In summary, our data revealed that type IV collagen controls the axogenesis of caudolateral GCs and RGCs by establishing and/or maintaining the integrity of the BM. The role of type IV collagen in BM integrity and axogenesis may provide insight into the etiology and pathology of Alport syndrome.

## Materials and Methods

### Ethics Statement

The animal work in this study was approved by Nagoya University Animal Experiment Committee (approval number: 2014020503, 2015022304) and was conducted in accordance with “Regulations on Animal Experiments in Nagoya University (Regulation No. 71, March 12, 2007)” and the “Guidelines for Proper Conduct of Animal Experiments (June 1, 2006, Science Council of Japan).

### Wild-type, Mutant, and Transgenic (Tg) Zebrafish Lines

Wild-type zebrafish (*Danio rerio*) with the Oregon AB genetic or TL background were used. In some experiments, mutant and Tg larvae were generated on the *casper* (*mitfa* ^*w2*^ *; roy* ^*a9*^) background [42]. The mutants used in this report were *sio* ^*rk18*^ [9] and *col4a5* ^*s510*^ (*dragnet*) [24]. To genotype *sio* ^*rk18*^, genomic DNA at the mutation site was amplified by PCR using the primers 5’-ATGTGTCCTGAGGGAATGACCAGG–3’ and 5’- TTCATTGGCGGTGCAGTAGAGGA–3’, followed by digestion with *Hph*I. To genotype *col4a5* ^*s510*^, genomic DNA at the mutation site was amplified by PCR using the primers 5’-GCCTGGTTCACCTGAGAAT–3’ and 5’-GATTGCCAGGTCATTTCCTT–3’, followed by digestion with *Nhe*I. The transgenic lines pou4f3:GAL4 (s311t), and pou4f3:GAL4, UAS:GAP-GFP (s318t) [34], and hsp70l:Slit2-GFP (rw015a) were previously reported [36]. The Gal4 trap lines gSA2AzGFF152B, gSAIGFF23C, and hspGFFDMC90A, which express a modified Gal4 (GFF) in GCs, were described previously [32]. gSA2AzGFF152B and hspGFFDMC90A express Gal4 in GCs of the Va, CCe, LCa, and EG, whereas gSAIGFF23C expresses Gal4 in GCs of the LCa and EG. The UAS:Kaede and UAS:GFP fish (rk8Tg and nkuasgfp1aTg in ZFIN: http://zfin.org/) were previously reported [43,44]. The zebrafish were maintained in an environmentally controlled room at the Bioscience and Biotechnology Center, Nagoya University.

### Single or Sparse Cell Labeling, Immunohistochemistry, Chemical Inhibitor, and Imaging

Tol1-mediated single-cell labeling was carried out as described previously [32]. Briefly, 5–25 pg of UAS:Kaede plasmid DNA and 50 pg of Tol1 transposase RNA were co-injected into 4-to-8-cell stage granule-cell-specific Gal4 Tg embryos. Larvae expressing Kaede in one or a few GCs were selected and observed under an epi-fluorescence microscope (MZ16A, Leica).

For immunostaining, anti-GFP (1:1000 dilution, rat, Nacalai), anti-parvalbumin 7 (Pvalb7, 1:1000, mouse monoclonal, ascites), anti-Vglut1 (1:1000, rabbit, affinity purified), anti-Neurod (1:500, mouse monoclonal, ascites) [9], anti-laminin (1:150, rabbit, Sigma), anti-phosphorylated FAK (anti-FAK[pY397], 1:200, rabbit, Life Technologies) antibodies were used. Immunostaining was performed as described previously [9,10]. Alexa Fluor 488 goat anti-rabbit and Alexa Fluor 555 goat anti-mouse IgG (H+L, Molecular Probes, Life Technologies) were used as secondary antibodies. To inhibit FAK, zebrafish were treated with FAK inhibitor PF–573228 (Sigma-Aldrich) in 1% dimethyl sulfoxide (DMSO) [45,46]. For single-cell labeling and immunohistochemistry, embryos and larvae were treated with 0.005% phenylthiourea from 12 hpf to prevent pigmentation. Optical clearing of some fixed samples was carried out with SeeDB reagent as previously reported [47,48].

Fluorescent images were captured with an LSM700 confocal laser-scanning microscope equipped with a 20x/0.8 numerical aperture (NA) or 40x/1.3 NA oil-immersion objective (Zeiss). To construct images, a series of optical sections was collected in the Z dimension (Z-stack) and projected as a single image or reconstructed in three dimensions to provide views of the image stack at different angles using the 3D projection program associated with the microscope (Zen, Zeiss) or by Imaris (Bitplane). The figures were constructed using Adobe Photoshop and Adobe Illustrator.

### Positional Cloning

The *sio* mutant was initially isolated from an AB strain [9]. *sio* ^*rk18*^ heterozygous fish were mated with TL fish to generate F1 families. Homozygous *sio* ^*rk18*^ mutant larvae were raised from the F1 crosses and selected by immunohistochemistry with the anti-Vglut1 antibody. We used samples of their genomic DNA for segregation analysis. Primers for the simple sequence length polymorphism (SSLP) markers used for positional cloning (Fig 2) were: 5’-TCATGTTGCTACAAGGCAAAA–3’ and 5’-TTGGGAAATGATTTGCAGTTT -3’ for 41.69 Mb, 5’-CAGAGGTCCTGATGGATTTGA–3’ and 5’-CAGACCCTGTGGAGGAAGAT–3’ for 42.5 Mb, 5’-CGCTCGTGGTGAGACAAATA–3’ and 5’-GGCGTCTGCATTGATGTTTA–3’ for 43.12 Mb, and 5’-AAGTCACATCTGGGTACGGC–3’ and 5’-TGCATCACTGAAAATGTGCA–3’ for 45.54 Mb (Z8584).

### Transmission Electron Microscopy (TEM) Analysis

Electron microscopic analysis was carried out as described previously [49,50] using JEM–1010 and JEM-1400Plus (JEOL).

### Laser Ablation

Laser ablation of the GFP-labeled GC axons was carried out using an LSM780-DUO-NLO laser-scanning inverted microscope (Zeiss) equipped with a Ti-sapphire femtosecond pulse laser (Chameleon Vision II, Coherent) as described previously [37]. A Ti-sapphire laser tuned to 880 nm was used for the ablation. Before and after laser irradiation, time-lapse images were captured with an LSM700 confocal laser-scanning microscope.

### Statistics

Statistic analyses are performed with Fisher’s exact test (S1, S2, S3 and S4 Tables) and Student’s t-test (S10 Fig).

## Supporting Information

S1 Fig Abnormal GC axons in GC-specific Gal4 line gSA2AzGFF152B in *sio* mutants. Labeling of the caudolateral GCs in 3-dpf (A-F) and 4-dpf (G-L) wild-type (WT, A-C, G-I) and *sio* ^*rk18*^ mutant (D-F, J-L) larvae with gSA2AzGFF152B; UAS:GFP (anti-GFP, green, A, C, D, F, G, I, J, L) and anti-Vglut1 (magenta, B, C, E, F, H, I, K, L) antibodies. Dorsal views with anterior to the left. Abnormal axons are indicated by arrows. Scale bars: 100 μm in L (applied to A-K).
(TIF)

S2 Fig Sparse cell labeling of GC axons in *col4a6* mutants. Sparse cell labeling was performed by injecting UAS:Kaede reporter DNA and Tol1 transposase RNA into the GC-specific Gal4 line hspGFFDMC90A. Immunostaning with anti-Kaede (A, B, D, E, G, H) and anti-Neurod antibodies (A, D, G). 5-dpf wild-type (A, B) and *col4a6* (D, E, G, H) larvae. Dorsal views. Arrowhead shows abnormal GC axons (E, H). (C, F, I) Schematic drawing of normal GC axons (C) and typical abnormal axons of GCs in LCa (I), EG and CCe (F). (J) Percentage of abnormal and normal GC axons in wild-type and the *col4a6* mutant larvae. Statistic analysis is shown in S1 Table. The GC axons were significantly affected in the *col4a6* mutant larvae, compared to wild-type larvae (Fisher’s exact test *p*<0.01 for LCa and EG, and *p*<0.05 for CCe). Most of the GC axons from the LCa and EG displayed misorientation, whereas only a portion of the GC axons in the CCe were affected. Scale bars: 50 μm in A (applied to B, D, E, G, H).
(TIF)

S3 Fig GC differentiation is not affected in *sio* mutants. Wild-type (A, B) and *sio* mutant (C, D) larvae were stained with anti-Neurod (white in A and C, magenta in B, D) and anti-Vglut1 (green, B, D) antibodies, which mark the nuclei and axons, respectively, of the GCs. Although the axons of the caudolateral GCs were affected in the *sio* mutants (marked by arrow in D), the expression of Neurod was not significantly affected in the mutants. Scale bars: 50 μm in D (applied to A-C).
(TIF)

S4 Fig Expression of *slit* and *robo* genes. Expression of *slit1a* (A, B), *slit1b* (C, D), *slit2* (E, F), *slit3* (G, H), *robo1* (I, J), *robo2* (K, L), *robo3* (M, N), and *robo4* (O, P) at 5 dpf. The expression was examined by whole-mount in situ hybridization. Lateral (A, C, E, G, I, K, M, O) and dorsal (B, D, F, H, J, L, N, P) views. *robo3* was expressed in the cerebellum (indicated by arrowheads, M, N). Scale bars: 200 μm in P (applied to A-O).
(TIF)

S5 Fig Overexpression of Slit2-GFP at 4 or 5 dpf does not affect GC axogenesis. Slit2 was overexpressed at 4 (C, D) or 5 dpf (E, F) by heat shock using the hsp70l:Slit2:GFP line. The resultant larvae were fixed at 5 dpf and stained with anti-parvalbumin7 (Pvalb7, green) and anti-Vglut1 (magenta) antibodies. (A, B) Non-induced control. Dorsal views. (B, D, F) High-magnification views of (A, C, E). Scale bars: 100 μm in E (applied to A, C); 40 μm in F (applied to B, D).
(TIF)

S6 Fig HNK–1 epitopes are sparsely distributed in the tectal BM region of *col4a6* mutants. Wild-type (A-D) and *col4a6* mutant (E-H) larvae were stained with anti-HNK–1 (zn12, A, B, D, E, F, H) and anti-GFP (A, C, D, E, G, H) antibodies. The RGC axons marked by pou4f3:Gal4; UAS:GAP-GFP in the tectal region; dorsal projection views (A, E) and lateral views (B-D, F-H). The HNK–1^+^ region was thinker in the tectal BM of the *col4a6* mutants (*n* = 5), compared to that in the wild type larvae (*n* = 3). The statistic analysis is shown in S4 Table. Scale bars: 20 μm in H (applied to A-G).
(TIF)

S7 Fig BM structure is disrupted in the col4a6 and col4a5 mutant tectum. Tectum of 5-dpf wild-type (G-I), *col4a6* mutant (A-F), and *col4a5* mutant (J-L) larvae was analyzed by electron microscopy. Cross sections (A, G, J). (B) Higher magnification view of box B in (A). (C, D) Higher magnification views of boxes C and D in (B). (E, F) Higher magnification views of boxes E and F in (D). (H) Higher magnification view of box H in (G). (I) Higher magnification view of box I in (H). (K) Higher magnification view of box K in (J). (L) Higher magnification view of box L in (K). The BM is indicated by yellow arrowheads. Truncation of the tectal BM was observed in both *col4a6* and *col4a5* mutants. Axons containing synaptic vesicles and microtubules are marked by blue and green dashed circles, respectively. Scale bars: 20 μm in A; 10 μm in B, G and J; 2 μm in C, O, H and K; 0.5 μm in E, F, I and L.
(TIF)

S8 Fig Abnormal GC axons are coupled with abnormal BM in the *col4a6* mutant hindbrain. The GC axons and BM structures in the hindbrain of wild-type (*n* = 2) and *col4a6* mutant larvae (*n* = 5) were analyzed as described in Fig 7. Typical examples were shown in Fig 7, and the rest of the samples (one) for WT and four for the mutant) are shown in this figure. Laminin–1 (A, E, I, M, Q), GFP (B, F, J, N, R), and merged images (C, G, K, O, S). Hindbrain BM and caudolateral GC axons are indicated by arrowheads and arrows, respectively. (D, H, L, P, T) Schematic representation of the BM (brown) and GC axons (green). GC axons that ran along the BM are indicated by closed triangles. GC axons that did not run along the BM are indicated by open triangles. Scale bars: 20 μm in S (applied to A-C, E-G, I-K, M-O, Q-R).
(TIF)

S9 Fig Laser ablation of GCs. Axons of caudolateral GCs in wild-type (2 larvae: a, b) and *col4a6* mutant (3 larvae: c-e) larvae that harbored hspGFFDMC90A; UAS:Kaede (a, c, d) or hspGFFDMC90A; UAS:GFP (b, e) transgenes were ablated by a laser at 5 dpf. The GC axons of the larvae were observed before (A, E, I, M, Q), soon after (B, F, J, N, R), or 1 (C, G, K, O, S) or 2 days (D, H, L, P, T) after the laser ablation. The experimental conditions were the same as described in the legend for Fig 8. More examples are shown in this figure. Dorsal views of the rostral hindbrain regions. The ablation points are indicated by arrows (B, F, J, N, R). Scale bars: 50 μm in T (applied to A-S).
(TIF)

S10 Fig Crest cells are not affected in *col4a6* mutants. Immunostaining of 5-dpf wild-type (A, Da-Dc) and *col4a6* mutant (B, Ea-Ec) larvae with anti-Vglut1 (A, B, Da, Dc, Ea, Ec) and anti-Pvalb7 (A, B, Db, Dc, Eb, Ec) antibodies. Higher-magnification images of boxes D and E in A (Da-Dc, Ea-Ec). The number of crest cells in each larva is indicated (C). The number of crest cell is not significantly different between wild-type and *col4a6* mutants (n.s., Student’s t-tests *p* = 0.504). Scale bars: 50 μm in A (applied to B), 20 μm in Da (applied to Db-Gc).
(TIF)

S11 Fig Expression of *ddr1*. Expression of *ddr1* (A-O) at 24 hpf (A, B, C), 2 dpf (D, E, F), 3 dpf (G, H, I), 4 dpf (J, K, L), and 5 dpf (M, N, O). The expression was examined by whole-mount in situ hybridization. Lateral (A, B, D, E, G, H, J, K, M, N) and dorsal (C, F, I, L, O) views. Cb: cerebellum region. Scale bars: 200 μm in A, D (applied to G), J, M: 100 μm in B (applied to C), 100 μm in E (applied to F, H, I, K, L, N, O).
(TIF)

S12 Fig Col4a6-independent FAK activation in GC axons. Immunostaining of 5-dpf wild-type (A-C) and *col4a6* (D-F) mutant larvae harboring the gSA2AzGFF152B; UAS:GFP transgene with anti-GFP (granule cell axons, A, C, D, F) and anti-phosphorylated FAK (B, C, E, F) antibodies. Dorsal views of the rostral hindbrain region. Note that the phosphorylated (active) form of FAK was similarly detected in the caudolateral GC axons in the wild-type and *col4a6* mutant hindbrain (marked by arrowheads). Scale bar: 100 μm in F (applied to A-E).
(TIF)

S13 Fig Inhibition of FAK does not affect formation of GC axons. Effect of an FAK inhibitor PF–573228 on axogenesis of GC axons. Wild-type larvae were untreated (A, B, 1% DMSO) or treated with 1 μM (C, D), 3 μM (E-H), 10 μM (I-L) and 30 μM (M-O) PF–5773228 (in 1% DMSO) from 10 hpf to 5 dpf. The resultant 5-dpf larvae were stained with anti-Vglut1 antibody. IC50 (half maximal inhibitory concentration) of PF–573228 is 30–100 nM [46] and 10 μM of PF–573228 was reported to sufficiently inhibit FAK in zebrafish embryos [45]. Scale bars: 100 μm in A (applied to B-O).
(TIF)

S1 Table Statistical analyses for GC axogenesis in *col4a6* mutants. Statistic analysis for S2 Fig. Single-cell analyses were performed by injecting UAS:Kaede reporter DNA into the GC-specific Gal4 line hspGFFDMC90A or gSA2AzGFF152B wild-type or the *sio* mutants. GC axons showing normal or abnormal (misoriented) structures were counted at 5 dpf. (A) Raw data. (B) Statistic analysis with Fisher’s exact test. The *p*-values are rounded off to three decimal place.
(DOCX)

S2 Table Statistic analysis for abnormal GC and RGC axons in *col4a6* and *col4a5*. Statistical analysis for Fig 3. 5-dpf wild-type, *col4a6*, and *col4a5* mutant larvae having normal or abnormal axons of caudolateral GCs and RGCs were counted. Statistic analysis was performed with Fisher’s exact test. Both the GC and RGC axons were affected in the *col4a6* and *col4a5* mutants (*p*<0.01). Significant differences in the abnormal GC and RGC axons between *col4a6* and *col4a5* mutants were not observed.
(DOCX)

S3 Table Statistic analysis for Slit2-GFP overexpression. Statistical analysis for Fig 4 and S5 Fig. Slit2 was overexpressed using the hsp70l:Slit2-GFP; pou4f3:Gal4 UAS:GAP43-GFP. Larvae showing normal or abnormal axons of GCs and RGCs were counted. The heat shock-mediated overexpression of Slit2-GFP induced abnormal axon projections of the RGCs. The overexpression of Slit2-GFP did not affect the GC axogenesis (Fisher’s exact test).
(DOCX)

S4 Table Statistic analysis of tectal BM staining. Statistic analysis for Fig 5 and S6 Fig. Larvae showing normal or abnormal BM staining of laminin–1 or zn12 in hindbrain or tectum regions were counted. For lamin–1 staining, larvae having truncated or split laminin–1 signals were considered as abnormal. For zn12 staining, larvae having zn12^+^ domains wider than 5 μm were considered as abnormal. Statistic analysis was performed with Fisher’s exact test. The *col4a6* mutation significantly affected deposition of laminin–1 (*p*<0.05 for hindbrain and tectum) and HNK–1 (*p*<0.01 for hindbrain, *p*<0.05 for tectum).
(DOCX)
