# Supplementary figures and images for: Type IV Collagen Controls the Axogenesis of Cerebellar Granule Cells by Regulating Basement Membrane Integrity in Zebrafish
Source: PLoS Genet. 2015 Oct 9;11(10):e1005587. doi: 10.1371/journal.pgen.1005587 (PMC4599943; doi:10.1371/journal.pgen.1005587)

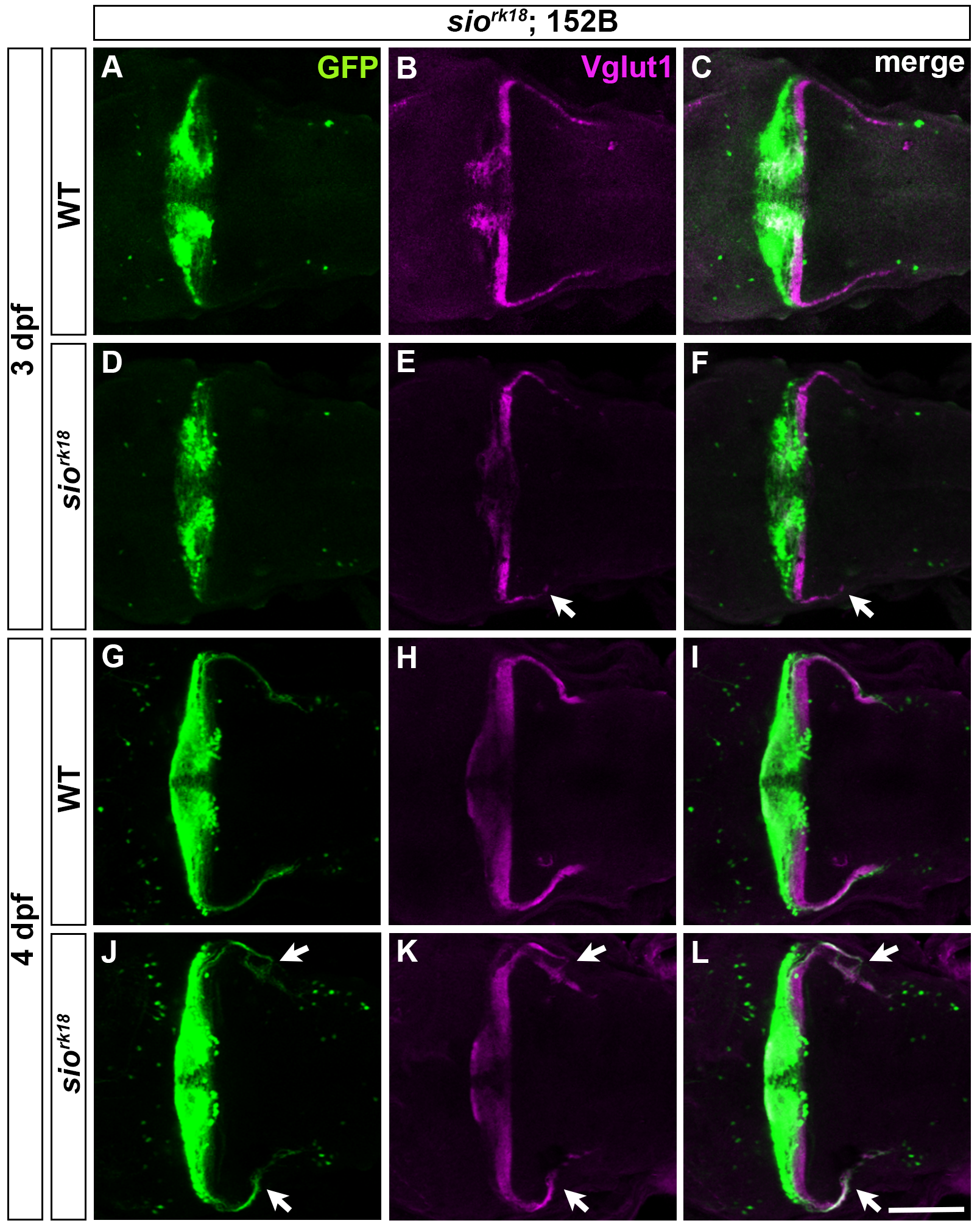

Supplement: S1 Fig — Labeling of the caudolateral GCs in 3-dpf (A-F) and 4-dpf (G-L) wild-type (WT, A-C, G-I) and sio rk18 mutant (D-F, J-L) larvae with gSA2AzGFF152B; UAS:GFP (anti-GFP, green, A, C, D, F, G, I, J, L) and anti-Vglut1 (magenta, B, C, E, F, H, I, K, L) antibodies. Dorsal views with anterior to the left. Abnormal axons are indicated by arrows. Scale bars: 100 μm in L (applied to A-K). (TIF) [file pgen.1005587.s001.tif]

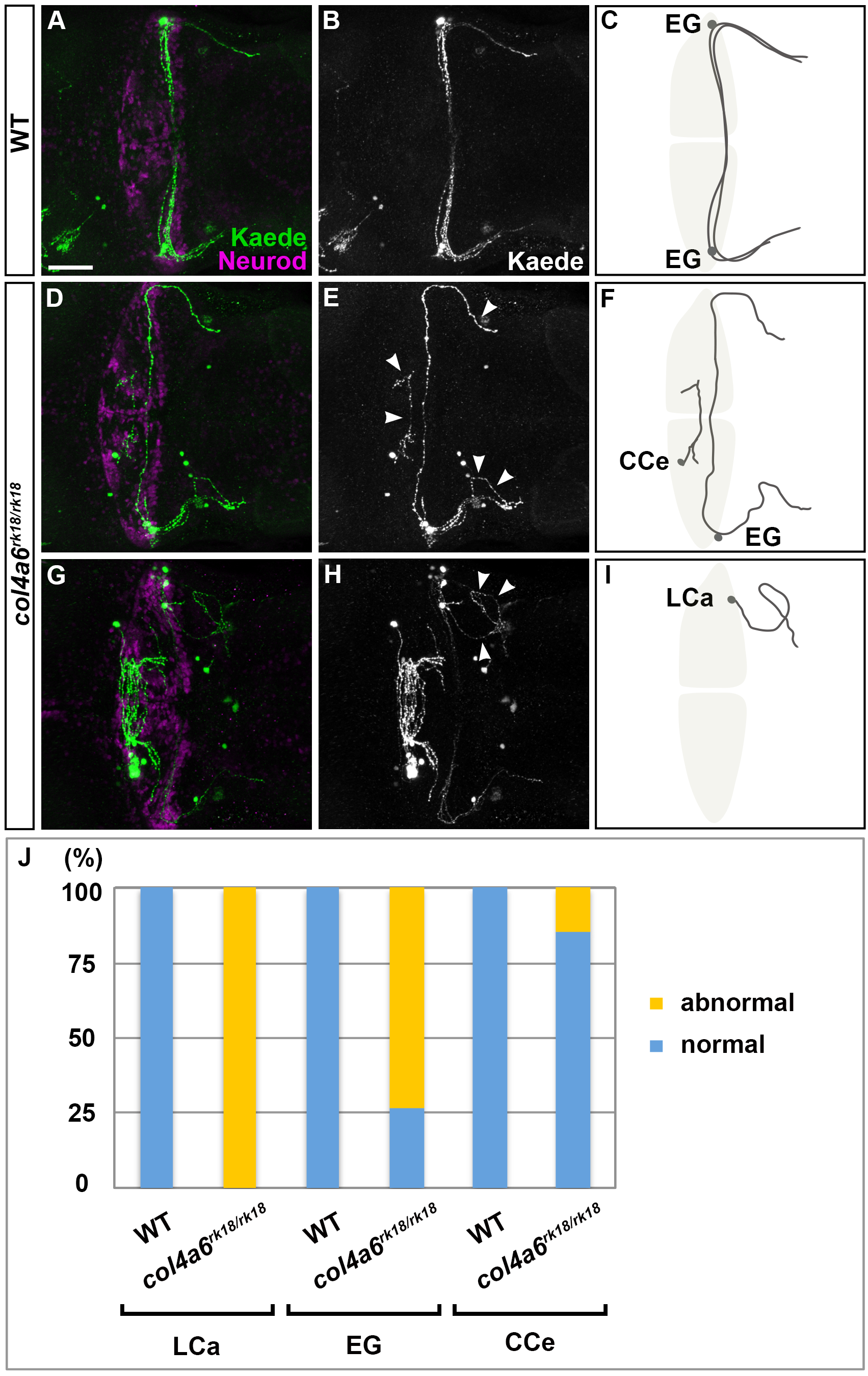

Supplement: S2 Fig — Sparse cell labeling was performed by injecting UAS:Kaede reporter DNA and Tol1 transposase RNA into the GC-specific Gal4 line hspGFFDMC90A. Immunostaning with anti-Kaede (A, B, D, E, G, H) and anti-Neurod antibodies (A, D, G). 5-dpf wild-type (A, B) and col4a6 (D, E, G, H) larvae. Dorsal views. Arrowhead shows abnormal GC axons (E, H). (C, F, I) Schematic drawing of normal GC axons (C) and typical abnormal axons of GCs in LCa (I), EG and CCe (F). (J) Percentage of abnormal and normal GC axons in wild-type and the col4a6 mutant larvae. Statistic analysis is shown in S1 Table. The GC axons were significantly affected in the col4a6 mutant larvae, compared to wild-type larvae (Fisher’s exact test p<0.01 for LCa and EG, and p<0.05 for CCe). Most of the GC axons from the LCa and EG displayed misorientation, whereas only a portion of the GC axons in the CCe were affected. Scale bars: 50 μm in A (applied to B, D, E, G, H). (TIF) [file pgen.1005587.s002.tif]

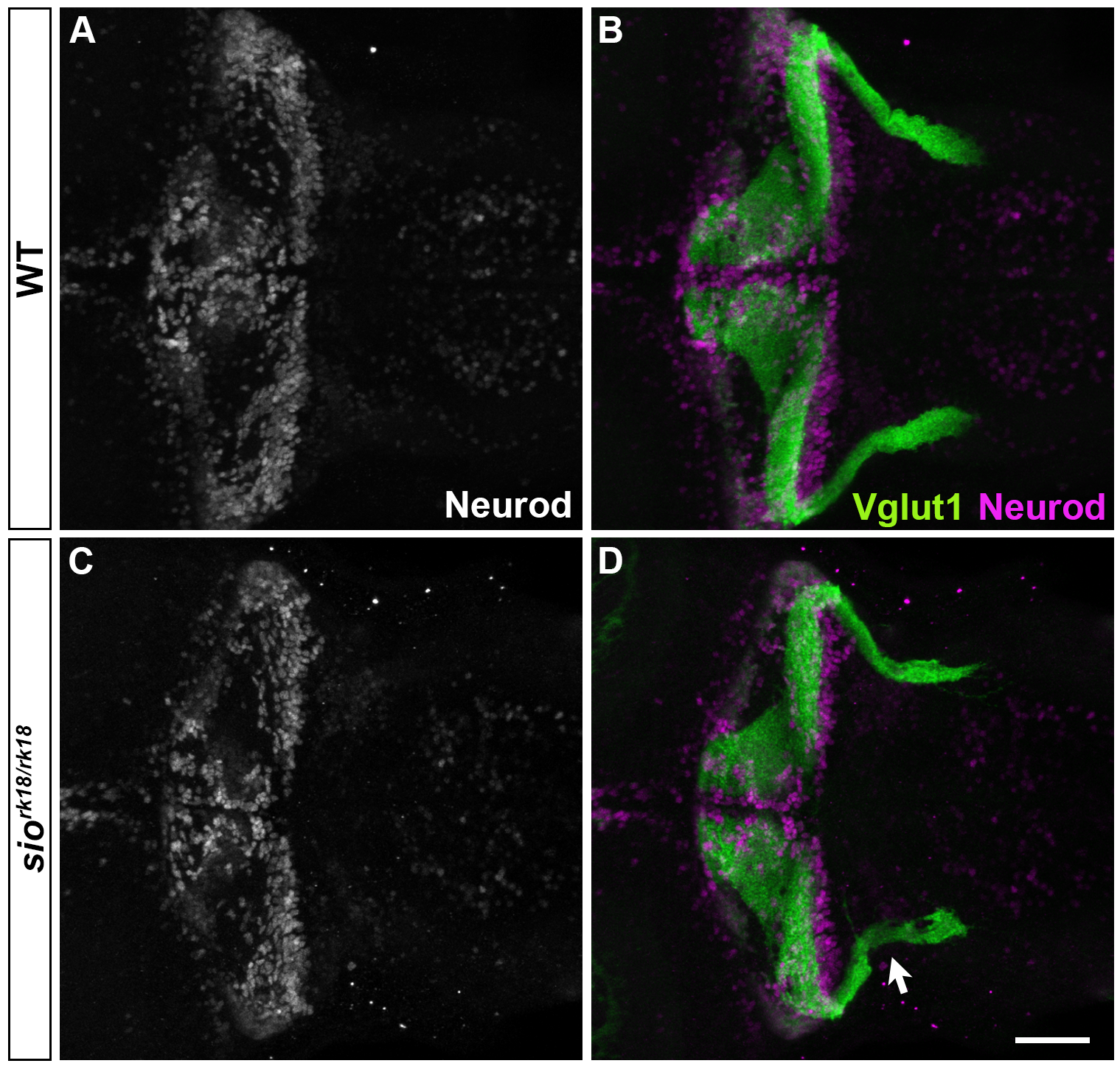

Supplement: S3 Fig — Wild-type (A, B) and sio mutant (C, D) larvae were stained with anti-Neurod (white in A and C, magenta in B, D) and anti-Vglut1 (green, B, D) antibodies, which mark the nuclei and axons, respectively, of the GCs. Although the axons of the caudolateral GCs were affected in the sio mutants (marked by arrow in D), the expression of Neurod was not significantly affected in the mutants. Scale bars: 50 μm in D (applied to A-C). (TIF) [file pgen.1005587.s003.tif]

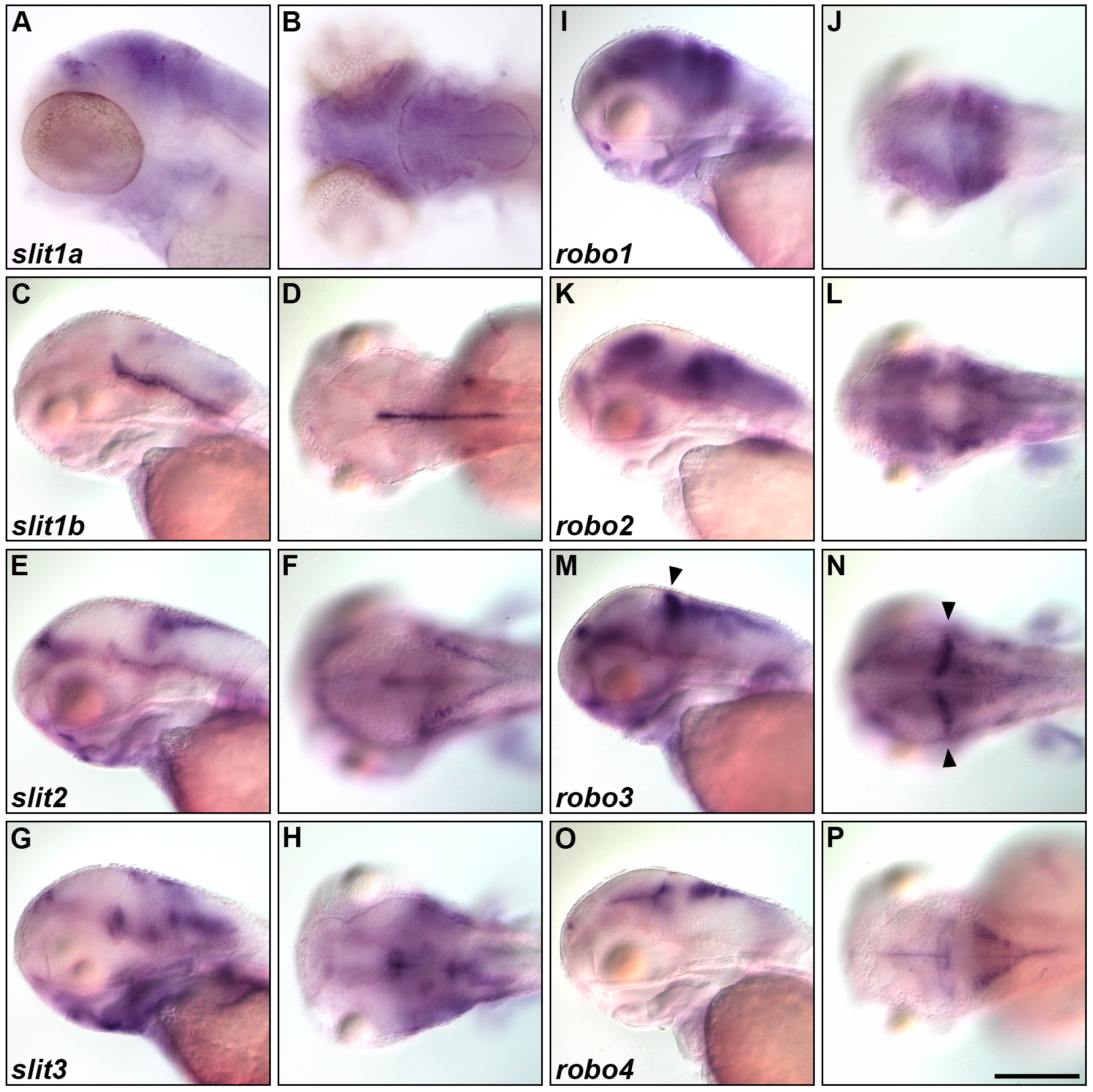

Supplement: S4 Fig — Expression of slit1a (A, B), slit1b (C, D), slit2 (E, F), slit3 (G, H), robo1 (I, J), robo2 (K, L), robo3 (M, N), and robo4 (O, P) at 5 dpf. The expression was examined by whole-mount in situ hybridization. Lateral (A, C, E, G, I, K, M, O) and dorsal (B, D, F, H, J, L, N, P) views. robo3 was expressed in the cerebellum (indicated by arrowheads, M, N). Scale bars: 200 μm in P (applied to A-O). (TIF) [file pgen.1005587.s004.tif]

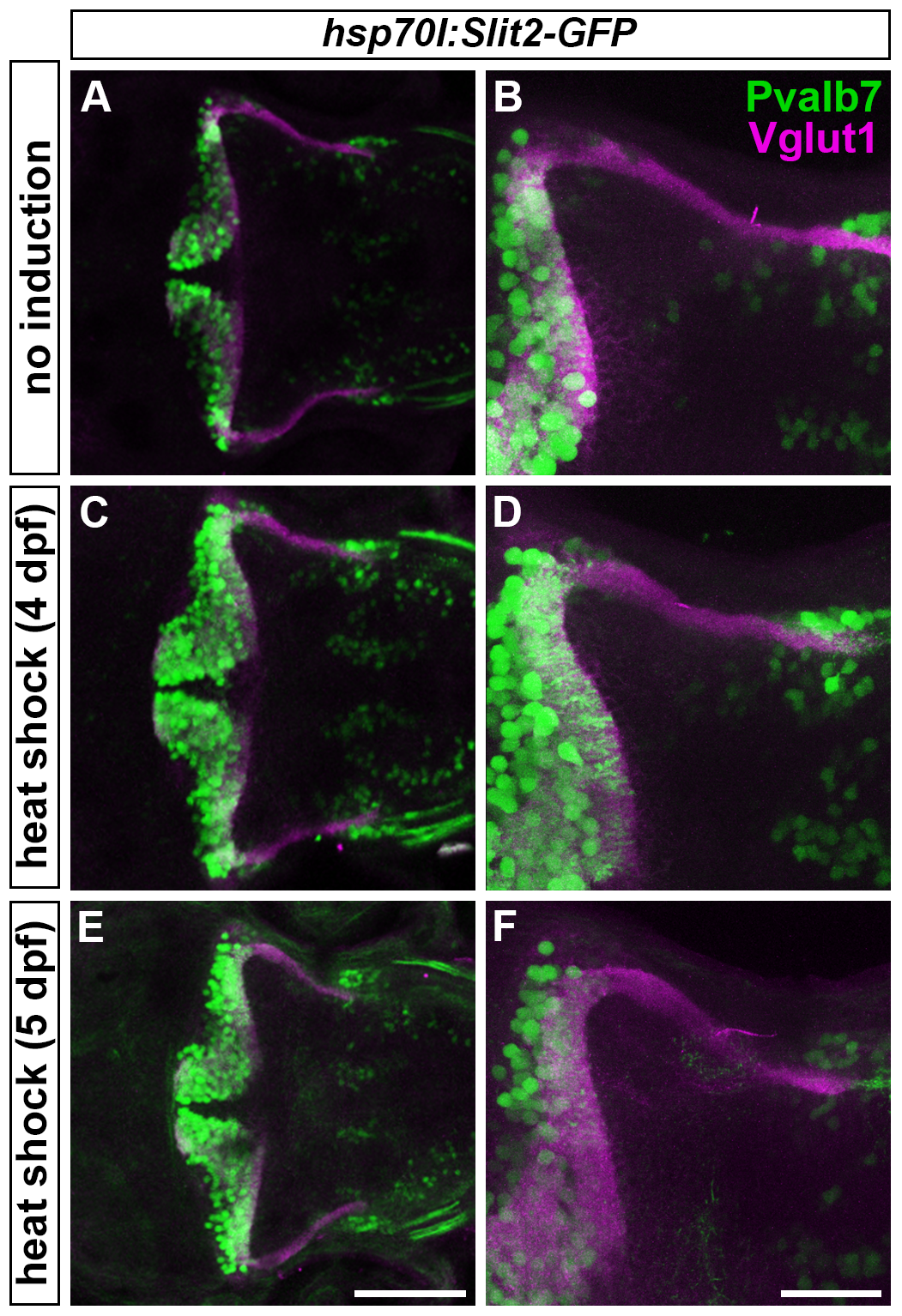

Supplement: S5 Fig — Slit2 was overexpressed at 4 (C, D) or 5 dpf (E, F) by heat shock using the hsp70l:Slit2:GFP line. The resultant larvae were fixed at 5 dpf and stained with anti-parvalbumin7 (Pvalb7, green) and anti-Vglut1 (magenta) antibodies. (A, B) Non-induced control. Dorsal views. (B, D, F) High-magnification views of (A, C, E). Scale bars: 100 μm in E (applied to A, C); 40 μm in F (applied to B, D). (TIF) [file pgen.1005587.s005.tif]

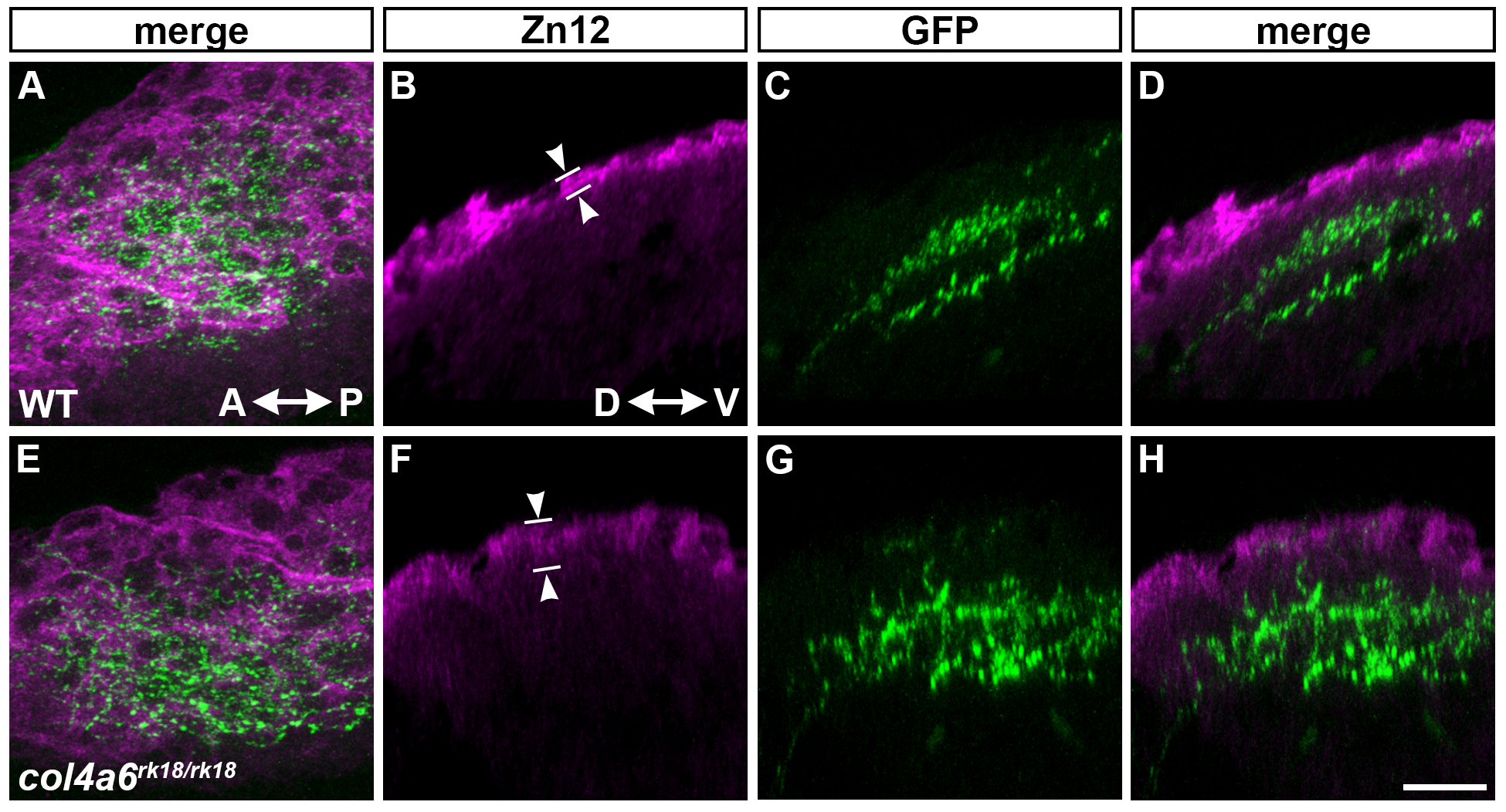

Supplement: S6 Fig — Wild-type (A-D) and col4a6 mutant (E-H) larvae were stained with anti-HNK–1 (zn12, A, B, D, E, F, H) and anti-GFP (A, C, D, E, G, H) antibodies. The RGC axons marked by pou4f3:Gal4; UAS:GAP-GFP in the tectal region; dorsal projection views (A, E) and lateral views (B-D, F-H). The HNK–1+ region was thinker in the tectal BM of the col4a6 mutants (n = 5), compared to that in the wild type larvae (n = 3). The statistic analysis is shown in S4 Table. Scale bars: 20 μm in H (applied to A-G). (TIF) [file pgen.1005587.s006.tif]

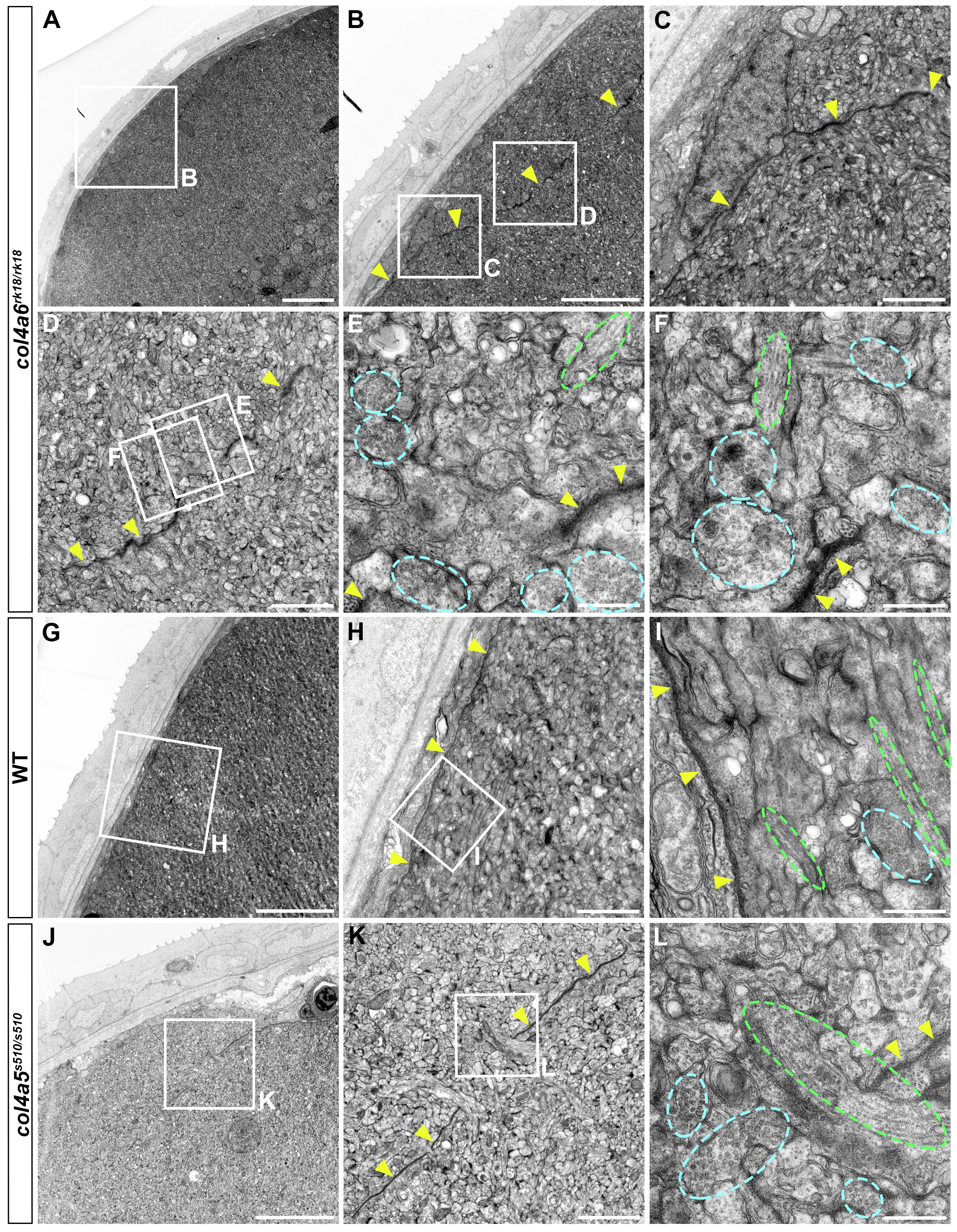

Supplement: S7 Fig — Tectum of 5-dpf wild-type (G-I), col4a6 mutant (A-F), and col4a5 mutant (J-L) larvae was analyzed by electron microscopy. Cross sections (A, G, J). (B) Higher magnification view of box B in (A). (C, D) Higher magnification views of boxes C and D in (B). (E, F) Higher magnification views of boxes E and F in (D). (H) Higher magnification view of box H in (G). (I) Higher magnification view of box I in (H). (K) Higher magnification view of box K in (J). (L) Higher magnification view of box L in (K). The BM is indicated by yellow arrowheads. Truncation of the tectal BM was observed in both col4a6 and col4a5 mutants. Axons containing synaptic vesicles and microtubules are marked by blue and green dashed circles, respectively. Scale bars: 20 μm in A; 10 μm in B, G and J; 2 μm in C, O, H and K; 0.5 μm in E, F, I and L. (TIF) [file pgen.1005587.s007.tif]

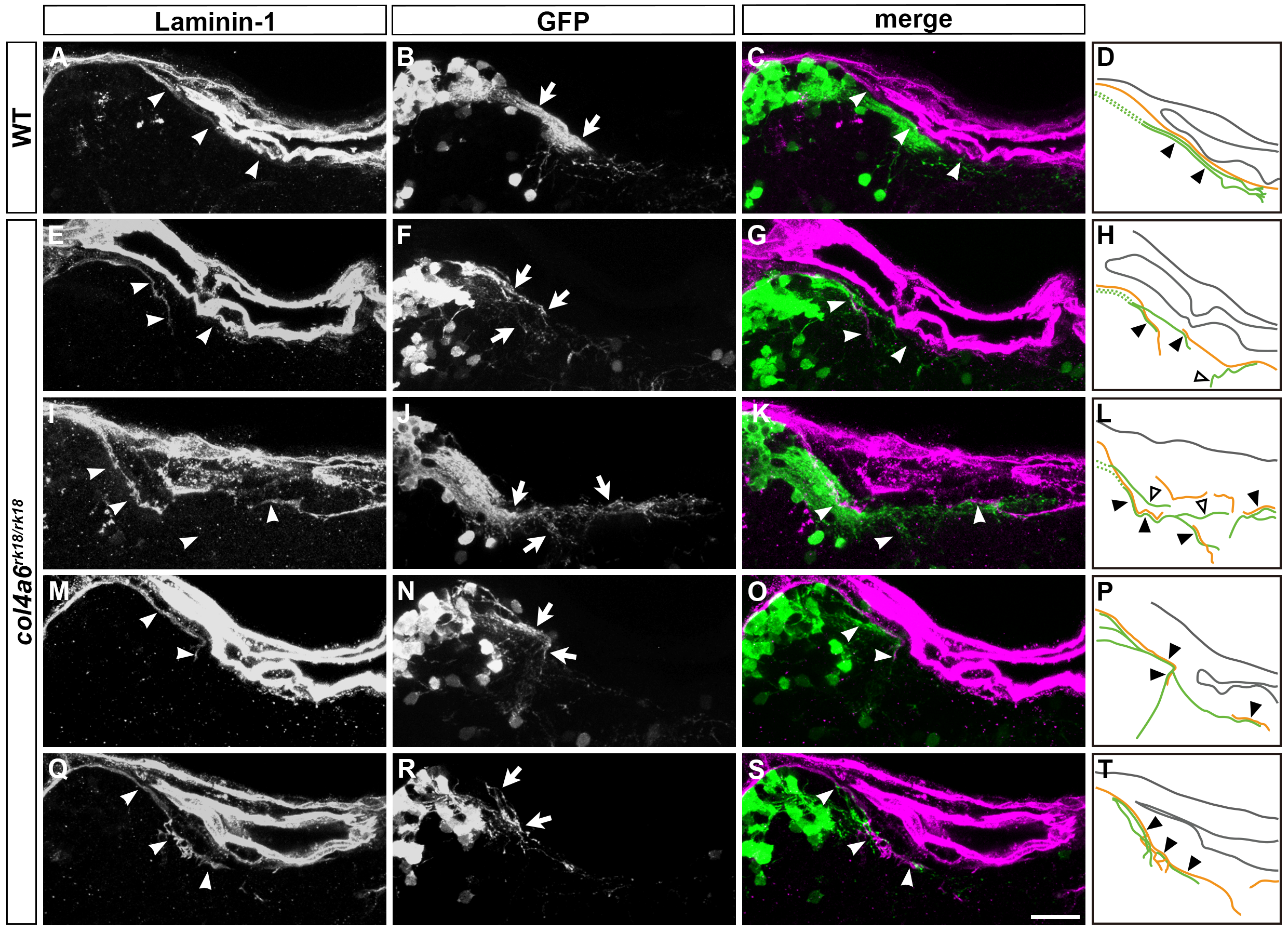

Supplement: S8 Fig — The GC axons and BM structures in the hindbrain of wild-type (n = 2) and col4a6 mutant larvae (n = 5) were analyzed as described in Fig 7. Typical examples were shown in Fig 7, and the rest of the samples (one) for WT and four for the mutant) are shown in this figure. Laminin–1 (A, E, I, M, Q), GFP (B, F, J, N, R), and merged images (C, G, K, O, S). Hindbrain BM and caudolateral GC axons are indicated by arrowheads and arrows, respectively. (D, H, L, P, T) Schematic representation of the BM (brown) and GC axons (green). GC axons that ran along the BM are indicated by closed triangles. GC axons that did not run along the BM are indicated by open triangles. Scale bars: 20 μm in S (applied to A-C, E-G, I-K, M-O, Q-R). (TIF) [file pgen.1005587.s008.tif]

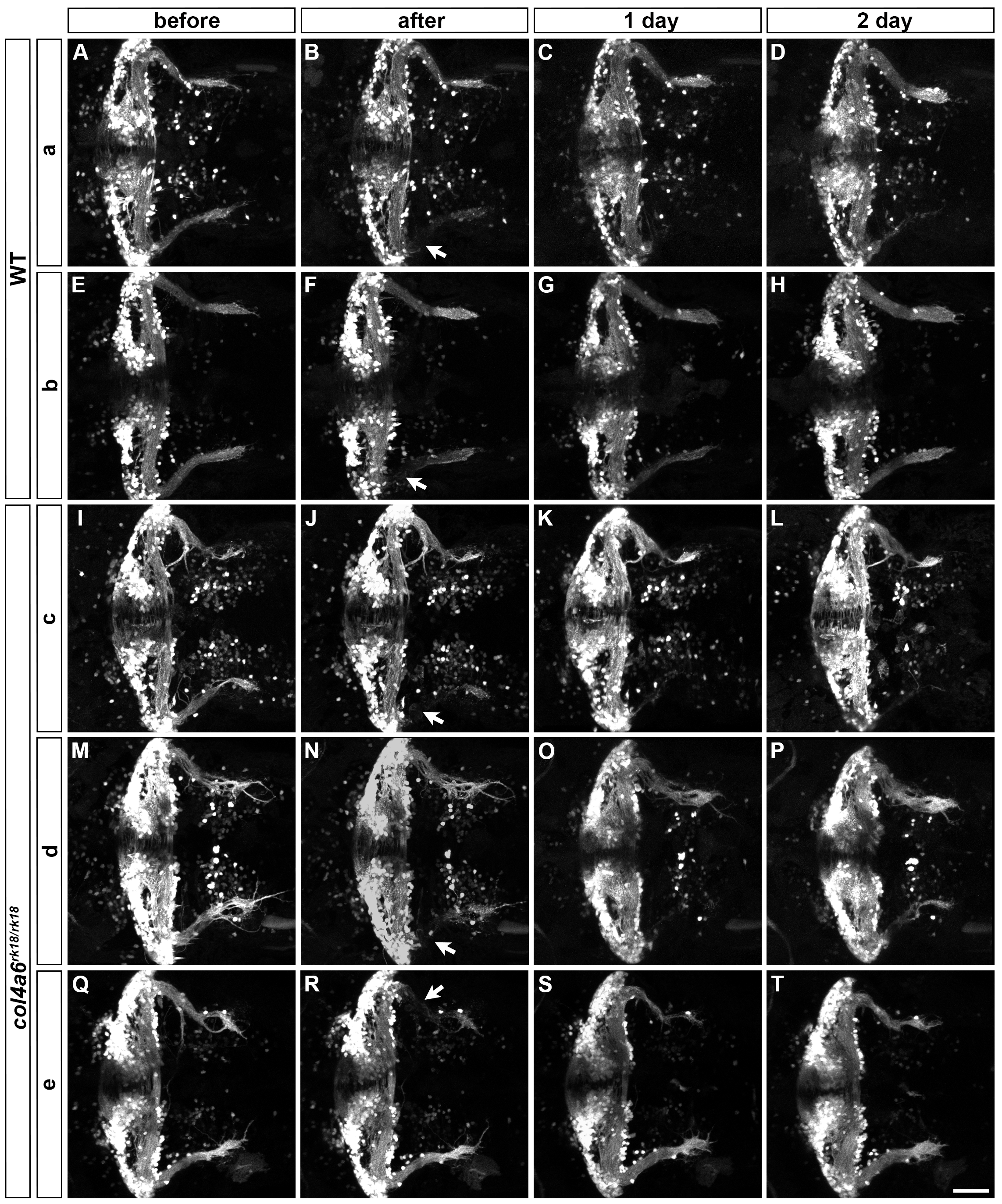

Supplement: S9 Fig — Axons of caudolateral GCs in wild-type (2 larvae: a, b) and col4a6 mutant (3 larvae: c-e) larvae that harbored hspGFFDMC90A; UAS:Kaede (a, c, d) or hspGFFDMC90A; UAS:GFP (b, e) transgenes were ablated by a laser at 5 dpf. The GC axons of the larvae were observed before (A, E, I, M, Q), soon after (B, F, J, N, R), or 1 (C, G, K, O, S) or 2 days (D, H, L, P, T) after the laser ablation. The experimental conditions were the same as described in the legend for Fig 8. More examples are shown in this figure. Dorsal views of the rostral hindbrain regions. The ablation points are indicated by arrows (B, F, J, N, R). Scale bars: 50 μm in T (applied to A-S). (TIF) [file pgen.1005587.s009.tif]

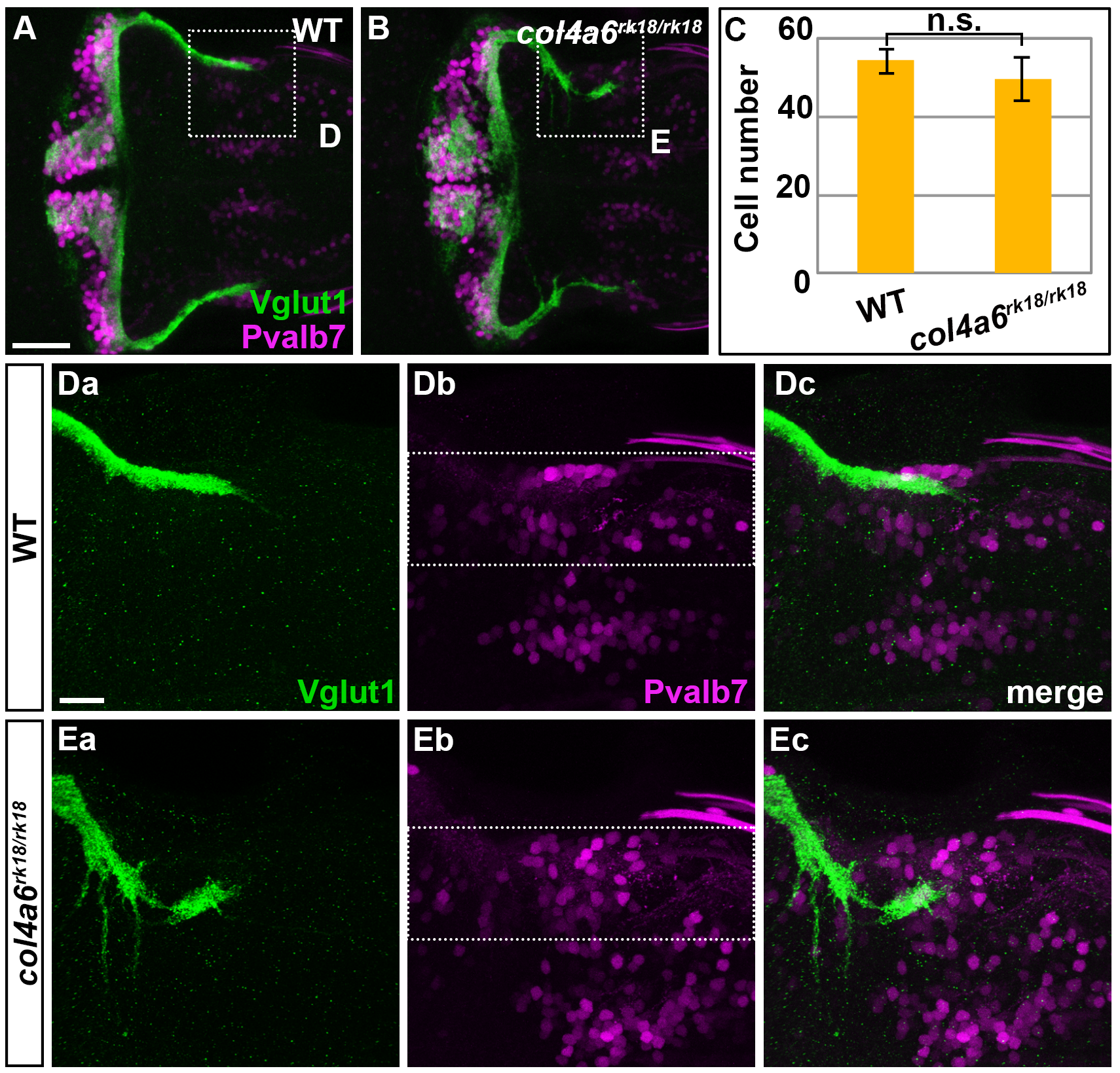

Supplement: S10 Fig — Immunostaining of 5-dpf wild-type (A, Da-Dc) and col4a6 mutant (B, Ea-Ec) larvae with anti-Vglut1 (A, B, Da, Dc, Ea, Ec) and anti-Pvalb7 (A, B, Db, Dc, Eb, Ec) antibodies. Higher-magnification images of boxes D and E in A (Da-Dc, Ea-Ec). The number of crest cells in each larva is indicated (C). The number of crest cell is not significantly different between wild-type and col4a6 mutants (n.s., Student’s t-tests p = 0.504). Scale bars: 50 μm in A (applied to B), 20 μm in Da (applied to Db-Gc). (TIF) [file pgen.1005587.s010.tif]

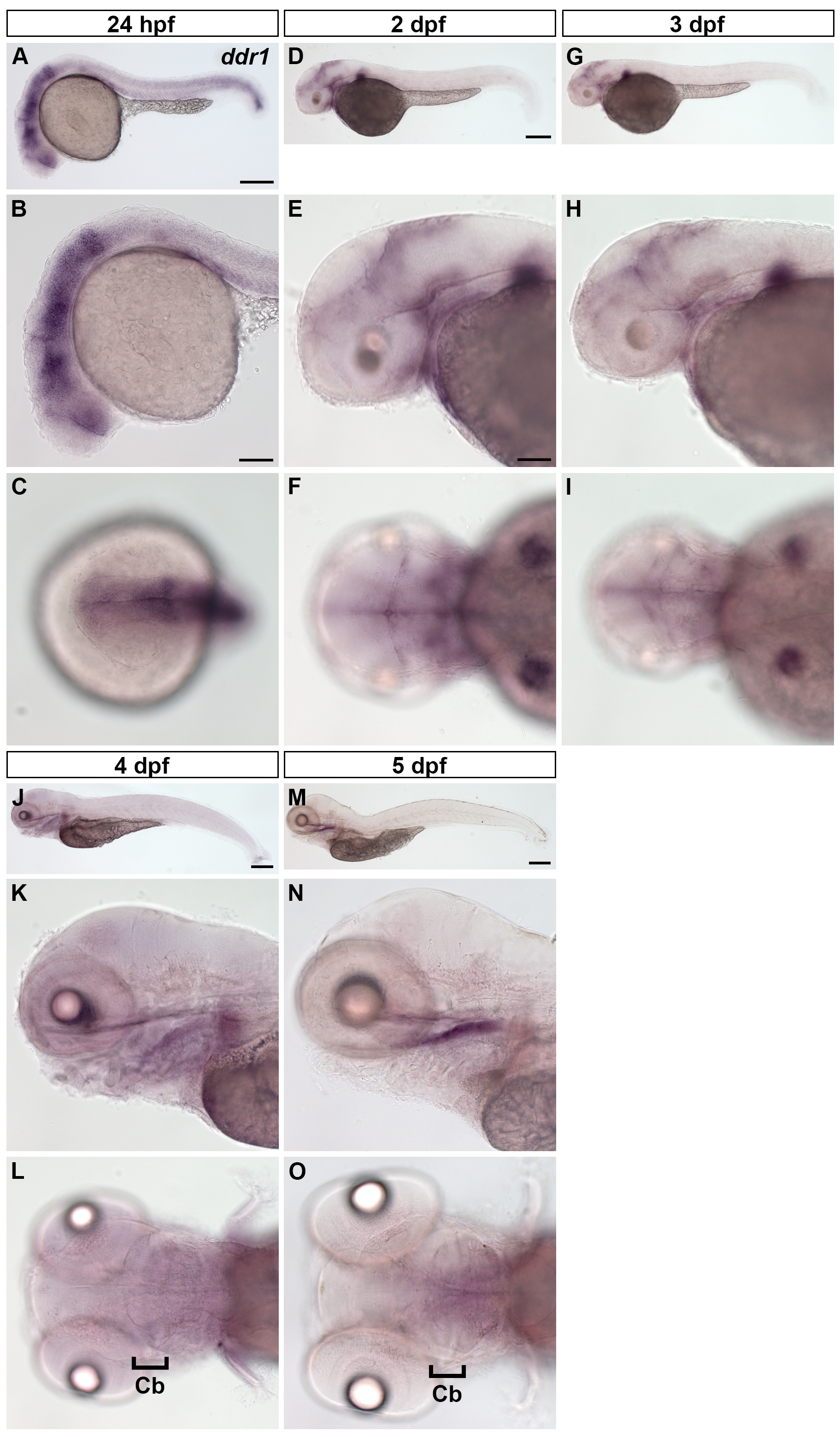

Supplement: S11 Fig — Expression of ddr1 (A-O) at 24 hpf (A, B, C), 2 dpf (D, E, F), 3 dpf (G, H, I), 4 dpf (J, K, L), and 5 dpf (M, N, O). The expression was examined by whole-mount in situ hybridization. Lateral (A, B, D, E, G, H, J, K, M, N) and dorsal (C, F, I, L, O) views. Cb: cerebellum region. Scale bars: 200 μm in A, D (applied to G), J, M: 100 μm in B (applied to C), 100 μm in E (applied to F, H, I, K, L, N, O). (TIF) [file pgen.1005587.s011.tif]

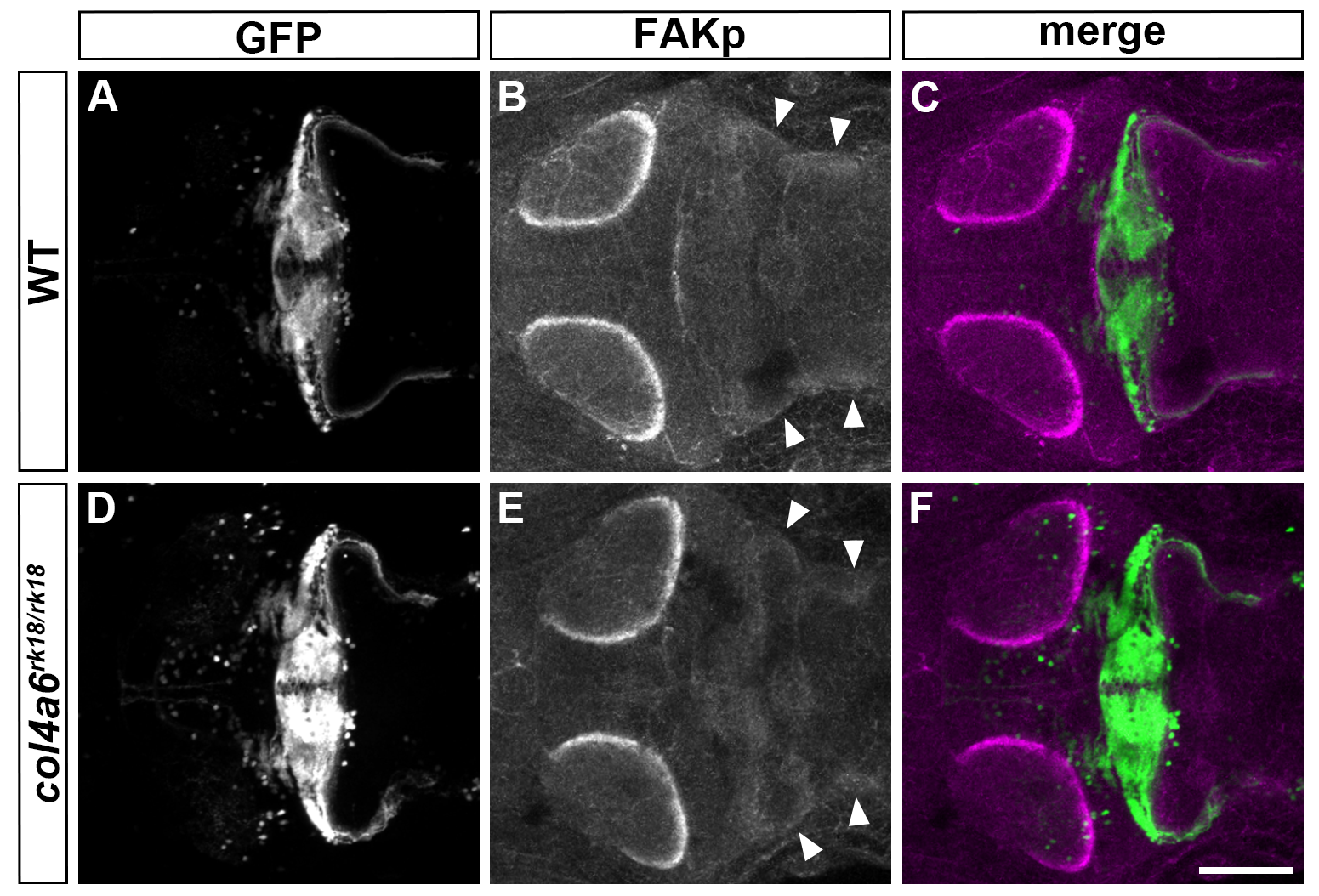

Supplement: S12 Fig — Immunostaining of 5-dpf wild-type (A-C) and col4a6 (D-F) mutant larvae harboring the gSA2AzGFF152B; UAS:GFP transgene with anti-GFP (granule cell axons, A, C, D, F) and anti-phosphorylated FAK (B, C, E, F) antibodies. Dorsal views of the rostral hindbrain region. Note that the phosphorylated (active) form of FAK was similarly detected in the caudolateral GC axons in the wild-type and col4a6 mutant hindbrain (marked by arrowheads). Scale bar: 100 μm in F (applied to A-E). (TIF) [file pgen.1005587.s012.tif]

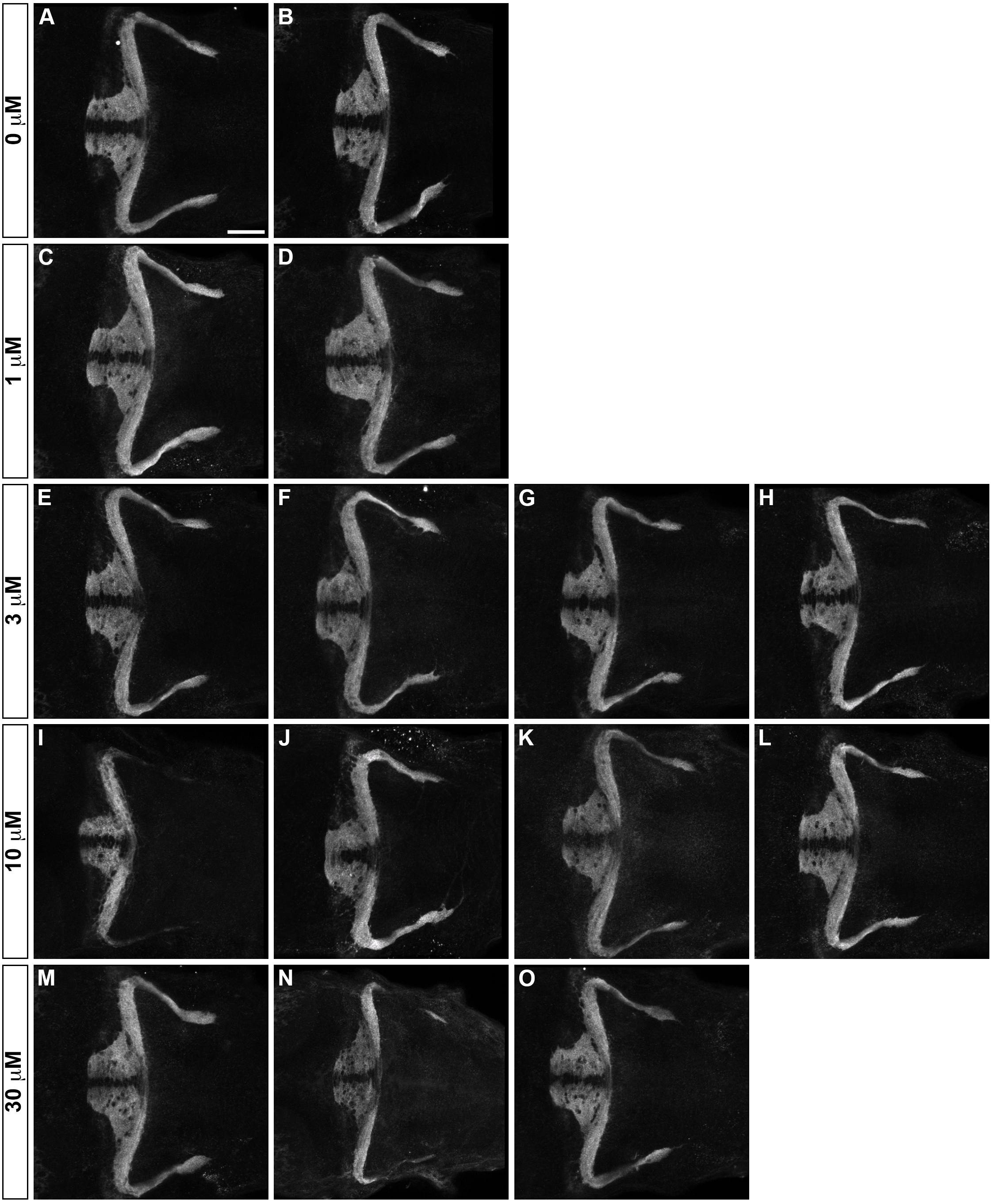

Supplement: S13 Fig — Effect of an FAK inhibitor PF–573228 on axogenesis of GC axons. Wild-type larvae were untreated (A, B, 1% DMSO) or treated with 1 μM (C, D), 3 μM (E-H), 10 μM (I-L) and 30 μM (M-O) PF–5773228 (in 1% DMSO) from 10 hpf to 5 dpf. The resultant 5-dpf larvae were stained with anti-Vglut1 antibody. IC50 (half maximal inhibitory concentration) of PF–573228 is 30–100 nM [46] and 10 μM of PF–573228 was reported to sufficiently inhibit FAK in zebrafish embryos [45]. Scale bars: 100 μm in A (applied to B-O). (TIF) [file pgen.1005587.s013.tif]
