# Supplementary material for: Type IV Collagen Controls the Axogenesis of Cerebellar Granule Cells by Regulating Basement Membrane Integrity in Zebrafish
Source: PLoS Genet. 2015 Oct 9;11(10):e1005587. doi: 10.1371/journal.pgen.1005587 (PMC4599943; doi:10.1371/journal.pgen.1005587)
Supplement: S1 Table — Statistic analysis for S2 Fig. Single-cell analyses were performed by injecting UAS:Kaede reporter DNA into the GC-specific Gal4 line hspGFFDMC90A or gSA2AzGFF152B wild-type or the sio mutants. GC axons showing normal or abnormal (misoriented) structures were counted at 5 dpf. (A) Raw data. (B) Statistic analysis with Fisher’s exact test. The p-values are rounded off to three decimal place. (DOCX) [file pgen.1005587.s014.docx]

(A) Raw data for single cell analyses

| Genotypes | | WT | | | | | |
| --- | --- | --- | --- | --- | --- | --- | --- |
| Granule cells | | LCa | | EG | | CCe | |
| Tracing | Larva | Normal | Abnormal | Normal | Abnormal | Normal | Abnormal |
| 90A; UAS:Kaede | #1 | 0 | 0 | 0 | 0 | 5 | 0 |
|  | #2 | 0 | 0 | 8 | 0 | 11 | 0 |
|  | #3 | 3 | 0 | 10 | 0 | 0 | 0 |
|  | #4 | 2 | 0 | 9 | 0 | 0 | 0 |
|  | #5 | 0 | 0 | 1 | 0 | 0 | 0 |
|  | #6 | 4 | 0 | 0 | 0 | 5 | 0 |
|  | #7 | 2 | 0 | 9 | 0 | 5 | 0 |
|  | #8 | 3 | 0 | 2 | 0 | 2 | 0 |
| 152B; UAS:Kaede | #9 | 0 | 0 | 0 | 0 | 5 | 0 |
|  | #10 | 0 | 0 | 3 | 0 | 0 | 0 |
|  | #11 | 0 | 0 | 0 | 0 | 13 | 0 |
| Genotypes | | *sio^rk18/rk18^* (*col4a6^rk18/rk18^*) | | | | | |
| Granule cells | | LCa | | EG | | CCe | |
| Tracing | Larva | Normal | Abnormal | Normal | Abnormal | Normal | Abnormal |
| 90A; UAS:Kaede | #12 | 0 | 0 | 1 | 1 | 0 | 1 |
|  | #13 | 0 | 0 | 0 | 3 | 1 | 0 |
|  | #14 | 0 | 1 | 0 | 1 | 5 | 2 |
|  | #15 | 0 | 0 | 1 | 1 | 0 | 0 |
|  | #16 | 0 | 1 | 0 | 1 | 3 | 1 |
|  | #17 | 0 | 1 | 0 | 0 | 0 | 0 |
|  | #18 | 0 | 0 | 1 | 4 | 10 | 1 |
| 152B; UAS:Kaede | #19 | 0 | 0 | 1 | 0 | 10 | 0 |

(B) Statistic analysis

a: Granule cells in LCa

|  | Normal | Abnormal | Sum |
| --- | --- | --- | --- |
| WT | 14 | 0 | 14 |
| *sio^rk18/rk18^* | 0 | 3 | 3 |
| Sum | 14 | 3 | 17 |

*p*=0.001

b: Granule cells in EG

|  | Normal | Abnormal | Sum |
| --- | --- | --- | --- |
| WT | 42 | 0 | 42 |
| *sio^rk18/rk18^* | 4 | 11 | 15 |
| Sum | 46 | 11 | 57 |

*p*=0.000

c: Granule cells in CCe

|  | Normal | Abnormal | Sum |
| --- | --- | --- | --- |
| WT | 46 | 0 | 46 |
| *sio^rk18/rk18^* | 29 | 5 | 34 |
| Sum | 75 | 5 | 80 |

*p*=0.012
