# Supplementary material for: Type IV Collagen Controls the Axogenesis of Cerebellar Granule Cells by Regulating Basement Membrane Integrity in Zebrafish
Source: PLoS Genet. 2015 Oct 9;11(10):e1005587. doi: 10.1371/journal.pgen.1005587 (PMC4599943; doi:10.1371/journal.pgen.1005587)
Supplement: S2 Table — Statistical analysis for Fig 3. 5-dpf wild-type, col4a6, and col4a5 mutant larvae having normal or abnormal axons of caudolateral GCs and RGCs were counted. Statistic analysis was performed with Fisher’s exact test. Both the GC and RGC axons were affected in the col4a6 and col4a5 mutants (p<0.01). Significant differences in the abnormal GC and RGC axons between col4a6 and col4a5 mutants were not observed. (DOCX) [file pgen.1005587.s015.docx]

| Cells | GC | | RGC | |
| --- | --- | --- | --- | --- |
|  | Normal | Abnormal | Normal | Abnormal |
| WT | 11 | 0 | 8 | 0 |
| *col4a6^rk18/rk18^* | 0 | 5 | 0 | 4 |
| *col4a5^s510/s510^* | 0 | 3 | 0 | 3 |

WT vs *col4a6*: *p*=0.000 (GC), *p*=0.002(RGC)

WT vs *col4a5*: *p*=0.003 (GC), *p*=0.006(RGC)

*col4a6* vs. *col4a5*: *p*=1.000 (GC), *p*=1.000 (RGC)
