# Supplementary material for: Type IV Collagen Controls the Axogenesis of Cerebellar Granule Cells by Regulating Basement Membrane Integrity in Zebrafish
Source: PLoS Genet. 2015 Oct 9;11(10):e1005587. doi: 10.1371/journal.pgen.1005587 (PMC4599943; doi:10.1371/journal.pgen.1005587)
Supplement: S3 Table — Statistical analysis for Fig 4 and S5 Fig. Slit2 was overexpressed using the hsp70l:Slit2-GFP; pou4f3:Gal4 UAS:GAP43-GFP. Larvae showing normal or abnormal axons of GCs and RGCs were counted. The heat shock-mediated overexpression of Slit2-GFP induced abnormal axon projections of the RGCs. The overexpression of Slit2-GFP did not affect the GC axogenesis (Fisher’s exact test). (DOCX) [file pgen.1005587.s016.docx]

| Cells | | GC | | RGC | |
| --- | --- | --- | --- | --- | --- |
| Heat shock | | Normal | Abnormal | Normal | Abnormal |
| Not treated (NT) |  | 5 | 0 | 2 | 0 |
| Heat shocked (HS) | 3 dpf | 10 | 0 | 0 | 2 |
|  | 4 dpf | 6 | 0 | ND | ND |
|  | 5 dpf | 5 | 0 | ND | ND |

NT vs HS (3 dpf): *p*=1.000 (GC)

NT vs HS (4 dfp): *p*=1.000(GC)

NT vs HS (5 dfp): *p*=1.000(GC)
