# Supplementary material for: Type IV Collagen Controls the Axogenesis of Cerebellar Granule Cells by Regulating Basement Membrane Integrity in Zebrafish
Source: PLoS Genet. 2015 Oct 9;11(10):e1005587. doi: 10.1371/journal.pgen.1005587 (PMC4599943; doi:10.1371/journal.pgen.1005587)
Supplement: S4 Table — Statistic analysis for Fig 5 and S6 Fig. Larvae showing normal or abnormal BM staining of laminin–1 or zn12 in hindbrain or tectum regions were counted. For lamin–1 staining, larvae having truncated or split laminin–1 signals were considered as abnormal. For zn12 staining, larvae having zn12+ domains wider than 5 μm were considered as abnormal. Statistic analysis was performed with Fisher’s exact test. The col4a6 mutation significantly affected deposition of laminin–1 (p<0.05 for hindbrain and tectum) and HNK–1 (p<0.01 for hindbrain, p<0.05 for tectum). (DOCX) [file pgen.1005587.s017.docx]

(A) laminin-1 staining in hindbrain region

|  | Normal | Abnormal | Sum |
| --- | --- | --- | --- |
| WT | 2 | 0 | 2 |
| *col4a6^rk18/rk18^* | 0 | 5 | 5 |
| Sum | 2 | 5 | 7 |

p=0.048

(B) HNK-1 (zn12) staining in hindbrain region

|  | Normal | Abnormal | Sum |
| --- | --- | --- | --- |
| WT | 6 | 0 | 6 |
| *col4a6^rk18/rk18^* | 0 | 6 | 6 |
| Sum | 6 | 6 | 12 |

*p*=0.001

(C) laminin-1 staining in tectum region

|  | Normal | Abnormal | Sum |
| --- | --- | --- | --- |
| WT | 5 | 0 | 5 |
| *col4a6^rk18/rk18^* | 0 | 3 | 3 |
| Sum | 5 | 3 | 8 |

p=0.018

(D) HNK-1 (zn12) staining in tectum region

|  | Normal | Abnormal | Sum |
| --- | --- | --- | --- |
| WT | 3 | 0 | 3 |
| *col4a6^rk18/rk18^* | 0 | 5 | 5 |
| Sum | 3 | 5 | 8 |

*p*=0.018
